# Supplementary material for: Identification of Circulating MiR-4651 as Novel Biomarker for Metabolic Dysfunction–Associated Steatotic Liver Disease
Source: Gastro Hep Adv. 2025 Oct 30;5(2):100839. doi: 10.1016/j.gastha.2025.100839 (PMC12756624; doi:10.1016/j.gastha.2025.100839)
Supplement: Supplementary data [file mmc1.pdf]

Supplementary Information

Identification of Circulating MiR-4651 as Novel Biomarker for Metabolic Dysfunction-Associated Steatotic Liver Disease

Mélanie Kirchmeyer, Anthoula Gaigneaux, Florence A. Servais, Anita Arslanow, Claudia Rubie, Markus Casper, Matthias Glanemann, María L Martínez-Chantar, Marcin Krawczyk, Frank Lammert, Iris Behrmann

Table of content:

Supplementary Materials and Methods……………………………...…….…………...…….. p. 2

Supplementary References…...…………………………………….……………………… p. 2

Supplementary Figure A1……………..………………...……………………….……………… p. 3

Supplementary Figure A2………………………...………….………………………………… p. 4

Supplementary Figure A3………………………………..…………………………….………... p. 5

Supplementary Table A1………………………………………………………………….……... p. 6

Supplementary Materials and Methods

qPCR Analysis of MiRNAs Isolated from Primary Human Liver Cells upon Stimulation with Hyper-IL-6

Primary healthy liver cells (named HepLux 5, 6, 7, and 8), derived from patients with colon cancer who underwent liver metastasis surgery, were received from the Department of Surgery of the Saarland University Hospital (Homburg, Germany). Human primary hepatocyte collection was approved by the respective ethics committees in Germany (Ethik-Kommission der Ärztekammer des Saarlands, 79/12) and Luxembourg (Comité National d'Éthique de Recherche, 201309/07), and signed statements of informed consent were obtained from all patients.

The handling and the culture of the cells have been described previously (1). Briefly, cells were maintained in HCM medium (HCM BulletKit Medium, Lonza, Basel, Switzerland) supplemented with 10% FBS and 100 µg/mL normocin. Six hours before the first stimulation, the HCM medium was replaced by serum-free hepatocyte high performance medium (Upcyte technologies, Hamburg, Germany), then kept untreated (Ct) or stimulated with 20 ng/mL hyper-IL-6 (a "designer cytokine" comprising IL-6 linked to the extracellular domain of IL-6Rα, (2)) for 6, 24, 48, and 72 h. Total RNA was extracted using the Column-zol RNA isolation kit (Enzymax LLC, Lexington, Kentucky, USA) according to the manufacturer's instructions. 500 ng of total RNA was reverse-transcribed and qPCR for miR-4651 and miR-4530 was run with QuantiTech primers (Qiagen, Venlo, The Netherlands), as well as for small RNAs used as miRNA normalizers (3 out of RNU1A, RNU5A, SCARNA17, and SNORD95).

Supplementary References

1. Kirchmeyer M, Servais FA, Hamdorf M, et al. Cytokine-mediated modulation of the hepatic miRNome: miR-146b-5p is an IL-6-inducible miRNA with multiple targets. J Leukoc Biol 2018;104:987-1002.
2. Fischer M, Goldschmitt J, Peschel C, et al. I. A bioactive designer cytokine for human hematopoietic progenitor cell expansion. Nat Biotechnol 1997;15:142-145.
3. Kirchmeyer M, Gaigneaux A, Servais FA, et al. Altered profiles of circulating cytokines in chronic liver diseases (NAFLD/HCC): Impact of the PNPLA3I148M risk allele. Hepatol Commun 2023;7.

Supplementary Figure A1

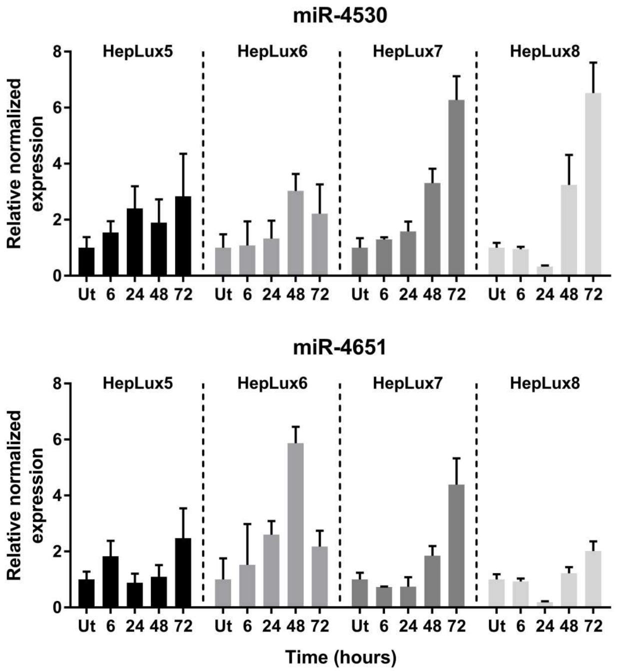

Supplementary Figure A1: Inducible expression of miR-4530 and miR-4651 in primary human hepatocytes treated with hyper-IL-6. Primary hepatocytes isolated from four patients (HepLux5-8) were stimulated with 20 ng/mL hyper-IL-6 for 6 - 72 h or left untreated (Ut). The levels of miR-4530 and miR-4651 in RNA extracts were determined by qPCR; results were normalized to the control. Error bars indicate the SD based on two technical replicates for the 6, 24, and 48 h time points, and three replicates for the Ut and 72 h conditions, all performed in parallel. (Please note: for HepLux8, one of the two 24 h-samples showed aberrant profiles and was therefore removed).

Supplementary Figure A2

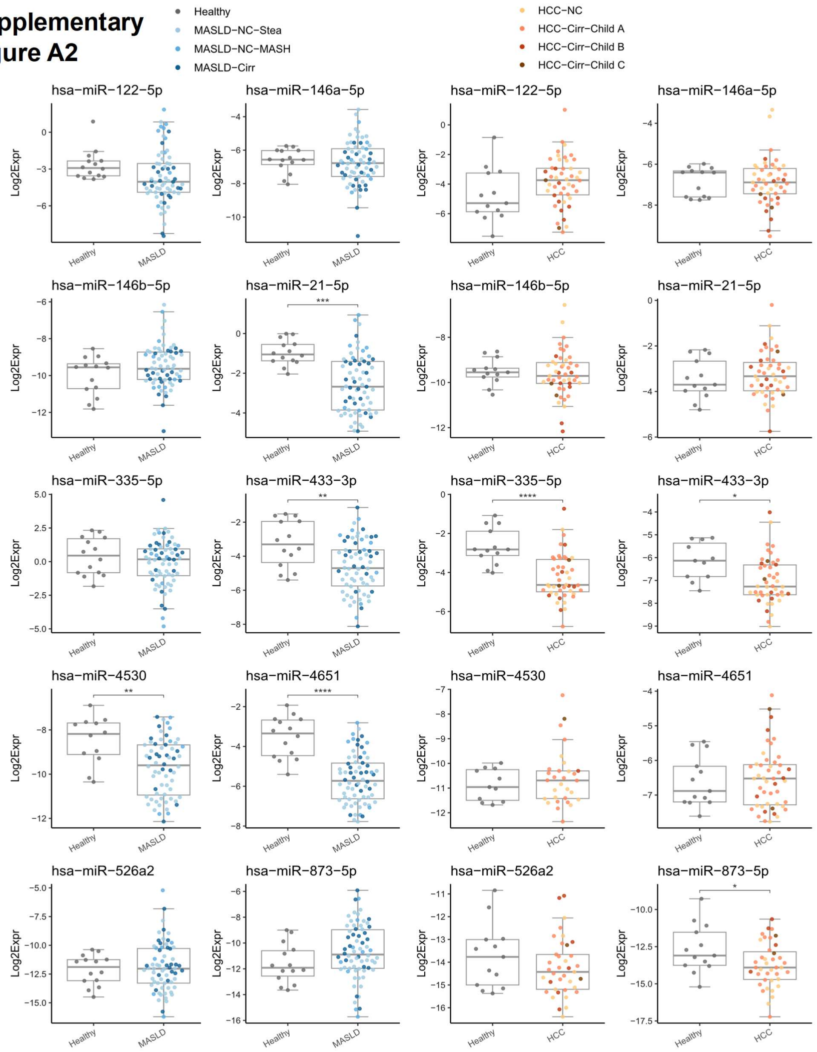

Supplementary Figure A2: Profiles of the 10 miRNAs in patients with MASLD and HCC *vs*. healthy controls. NAFLD and HCC samples were analyzed without further subdivision in disease-subtypes (in contrast to Figure 1). The horizontal line within the box plots represents the median, vertical lines from the boxes (whiskers) indicate the variability outside the upper and lower quartiles. Pairwise t-tests were performed, and p-values were adjusted for each miRNA using the Holm method. ****: $p < 0.0001$, ***: $p < 0.001$, **: $p < 0.01$, *: $p < 0.05$.

Supplementary Figure A3

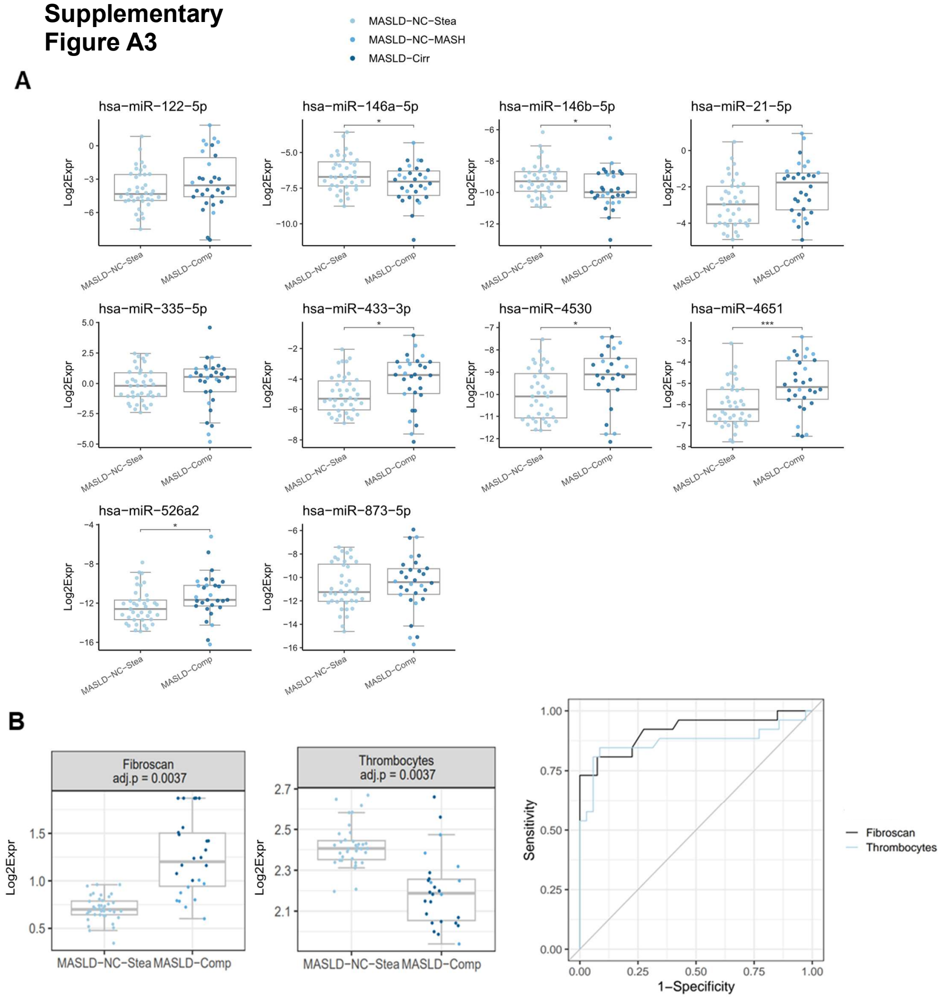

Supplementary Figure A3: Comparison of patients with uncomplicated MASLD (MASLD-NC-Stea, i.e., simple steatosis) *vs*. complicated MASLD (MASLD-Comp, i.e., MASH and cirrhosis). A) Profiles of the 10 miRNAs. B) Single-parameter ROC curves for "Fibroscan" and "Thrombocytes" (see also Figure 5 and Table S1). The BH-adjusted p-value of the GLM model is indicated.

Supplementary Table A1: Information about clinical parameters and data availability.

Parameters	Healthy	MASLD-NC-Stea	MASLD-NC-MASH	MASLD-Cirr	HCC-NC	HCC-Cirr	Overall
	(N=14)	(N=40)	(N=9)	(N=21)	(N=15)	(N=32)	(N=131*)
**Thrombocytes $(10^9/L)$**							
Mean (SD)	225 (58.4)	271 (71.6)	191 (67.3)	166 (92.2)	/	/	228 (85.8)
Median	216	255	177	141	/	/	223
[Min, Max]	[157, 338]	[157, 467]	[87.0, 298]	[97.0, 458]			[87.0, 467]
Missing	0 (0%)	5 (12.5%)	2 (22.2%)	2 (9.5%)			9 (10.7%*)
**GGT (U/L)**							
Mean (SD)	27.1 (22.8)	72.3 (73.9)	327 (440)	134 (105)	169 (144)	301 (423)	194 (304)
Median	19.5	39.0	154	125	121	200	109
[Min, Max]	[7.00, 97.0]	[21.0, 157]	[58.0, 1200]	[26.0, 438]	[24.0, 530]	[25.0, 2260]	[7.00, 2260]
Missing	0 (0%)	37 (92.5%)	3 (33.3%)	5 (23.8%)	1 (6.7%)	2 (6.3%)	48 (36.6%)
**AST (U/L)**							
Mean (SD)	20.1 (5.53)	32.3 (12.5)	92.3 (58.5)	46.0 (39.6)	39.2 (13.6)	63.7 (43.9)	49.2 (40.2)
Median	19.0	38.0	91.0	33.0	36.0	49.0	36.0
[Min, Max]	[12.0, 32.0]	[18.0, 41.0]	[32.0, 183]	[21.0, 173]	[20.0, 61.0]	[26.0, 205]	[12.0, 205]
Missing	1 (7.1%)	37 (92.5%)	3 (33.3%)	5 (23.8%)	4 (26.7%)	9 (28.1%)	59 (45.0%)
**Cytokine data**							
Available	11 (78.6%)	40 (100%)	8 (88.9%)	21 (100%)	6 (40.0%)	15 (46.9%)	101 (77.1%)
Missing	3 (21.4%)	0 (0%)	1 (11.1%)	0 (0%)	9 (60.0%)	17 (53.1%)	30 (22.9%)

For the six groups (Healthy, MASLD-NC-Stea, MASLD-NC-MASH, MASLD-Cirr, HCC-NC, HCC-Cirr), information about the clinical parameters "thrombocytes", GGT, and AST are indicated as mean ± SD and median. Moreover, the numbers of samples analyzed previously regarding concentrations of 22 cytokines (3) are indicated as well as the numbers of missing values.
*For "thrombocytes", no data were collected for HCC patients. Thus, the percentage was calculated only for the healthy and MASLD samples (N=84).
