# Supplementary material for: Identification of Circulating MiR-4651 as Novel Biomarker for Metabolic Dysfunction–Associated Steatotic Liver Disease
Source: Gastro Hep Adv. 2025 Oct 30;5(2):100839. doi: 10.1016/j.gastha.2025.100839 (PMC12756624; doi:10.1016/j.gastha.2025.100839)
Supplement: Extended PDF [file mmc2.pdf]

Gastro Hep Advances 2026;5:100839

ORIGINAL RESEARCH—CLINICAL

Identification of Circulating MiR-4651 as Novel Biomarker for Metabolic Dysfunction–Associated Steatotic Liver Disease

Mélanie Kirchmeyer,[1] Anthoula Gaigneaux,[1] Florence A. Servais,[1] Anita Arslanow,[2,3] Claudia Rubie,[4] Markus Casper,[2] Matthias Glanemann,[4] María L. Martínez-Chantar,[5,6] Marcin Krawczyk,[2,7] Frank Lammert,[2,8] and Iris Behrmann[1]

[1]Department of Life Sciences and Medicine, University of Luxembourg, Esch-sur-Alzette, Luxembourg; [2]Department of Medicine II, Saarland University Medical Center, Homburg, Germany; [3]Department of Gastroenterology, Hepatology, Infectious Diseases and Endocrinology, Hannover Medical School MHH, Hannover, Germany; [4]Department of Surgery, Saarland University Medical Center, Homburg, Germany; [5]Liver Disease Lab, Center for Cooperative Research in Biosciences (CIC bioGUNE), Basque Research and Technology Alliance, Derio, Spain; [6]Centro de Investigación Biomédica en Red de Enfermedades Hepáticas y Digestivas (CIBERehd), Carlos III National Health Institute, Madrid, Spain; [7]Department of Gastroenterology, Hepatology and Transplant Medicine, Medical Faculty, University of Duisburg-Essen, Essen, Germany; and [8]Center for Health Research Economics Hannover (CHERH), Hannover Medical School (MHH), Hannover, Germany.

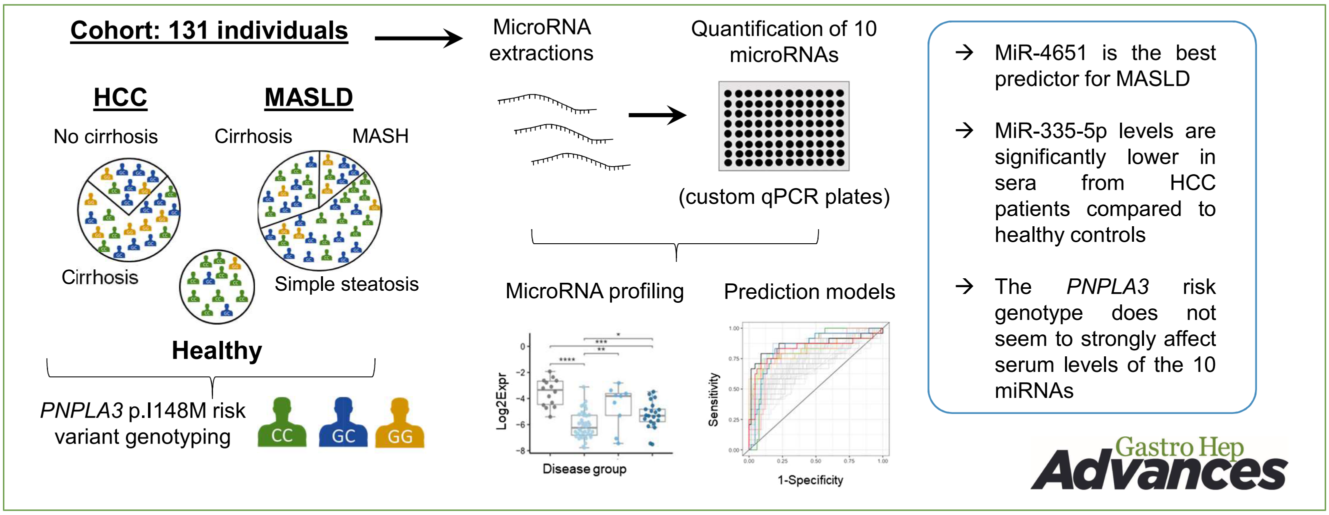

BACKGROUND AND AIMS: Metabolic dysfunction–associated steatotic liver disease (MASLD) affects >30% of adults and is becoming one of the leading causes of hepatocellular carcinoma (HCC). Reliable biomarkers are needed for the early diagnosis of HCC and detection of chronic liver diseases, like MASLD. Here we assessed the biomarker potential of circulating microRNAs in a cohort of patients genotyped for the risk allele of *patatin-like phospholipase domain-containing protein 3* (*PNPLA3*), associated with increased susceptibility to chronic liver diseases. **METHODS:** The cohort comprised 70 MASLD patients (40 with simple steatosis, 9 with metabolic dysfunction–associated steatohepatitis (MASH), 21 with cirrhosis), 47 HCC patients (32 with cirrhosis), and 14 healthy controls. Serum levels of miR-122-5p, miR-146a-5p, miR-146b-5p, miR-21-5p, miR-335-5p, miR-433-3p, miR-4530, miR-4651, miR-526a2, and miR-873-5p were quantified using custom qPCR plates. Their suitability for prediction of MASLD (steatosis/MASH/cirrhosis) or HCC was assessed by receiver operating characteristic curve analyses. **RESULTS:** MiR-4651 and miR-21-5p were significantly reduced in sera from patients with MASLD, particularly in those with simple steatosis. Both microRNAs effectively distinguished MASLD patients with simple steatosis from healthy controls (area under the curve: 0.95 and 0.89, respectively). Moreover, miR-4651 emerged as the best predictor for differentiating "complicated" MASLD (i.e., MASH or cirrhosis) from simple steatosis; the predictive values could be increased by including additional parameters into the models (Fibroscan,

Abbreviations used in this paper: **AUC, area under the curve; BH, Benjamini-Hochberg; HCC, hepatocellular carcinoma; MASH, metabolic dysfunction–associated steatohepatitis; MASLD, metabolic dysfunction–associated steatotic liver disease; MASLD-Comp, MASLD complication; miRNA, microRNA; NC, no cirrhosis; PNPLA3, Patatin-like phospholipase domain-containing protein 3; ROC, Receiver operating characteristic; Stea, steatosis; WT, wild-type.**

Most current article

Copyright © 2025 The Authors. Published by Elsevier Inc. on behalf of the AGA Institute. This is an open access article under the CC BY license (http://creativecommons.org/licenses/by/4.0/).
2772-5723
https://doi.org/10.1016/j.gastha.2025.100839

thrombocytes, cytokines, or other miRNAs). miR-335-5p showed strong ability to differentiate HCC from healthy individuals (area under the curve: 0.86). The *PNPLA3* p.I148M genotype was not associated with altered levels of microRNAs. **CONCLUSION:** Serum microRNAs, in particular miR-4651, may serve as additional biomarkers in patients with steatotic liver disease.

Keywords: circulating microRNA; hepatocellular carcinoma; liver cirrhosis; patatin-like phospholipase domain-containing protein 3; receiver operating characteristic curve analysis

Introduction

Metabolic dysfunction–associated steatotic liver disease (MASLD), the hepatic form of the metabolic syndrome, is a global challenge affecting approximately a third of the world population. While the earliest stage of MASLD, simple steatosis, has usually a benign course, in some patients it progresses to an inflammatory disease, namely metabolic dysfunction–associated steatohepatitis (MASH). MASH can lead to fibrosis and cirrhosis and increases the risk of developing hepatocellular carcinoma (HCC)[1] which is the most common form of primary liver cancer. Recently MASLD has been recognized as the most rapidly growing cause of HCC.[2] Next to lifestyle and environmental factors, several genes, such as *patatin-like phospholipase domain-containing protein 3 (PNPLA3)*, affect the risk of chronic liver diseases and HCC. As the early stages of HCC are usually asymptomatic, it is often detected at an advanced stage.[3] In this context, biomarkers from liquid biopsies hold promise for detecting HCC at earlier stages.

MicroRNAs (miRNAs), short noncoding RNA molecules (20–24 nucleotides), bind to specific mRNA targets, thereby influencing gene expression by inhibition of translation and/or degradation of mRNA. Apart from their intracellular roles, miRNAs can be found in all body fluids. Circulating miRNAs offer several advantages as biomarkers: they are stable, show considerable tissue-specificity, it is possible to quantitatively assess them, and their profiles change during diseases. Thus, there is considerable interest in miRNAs as possible diagnostic biomarkers for various diseases, including HCC and MASLD, either alone or in combination with other disease-associated parameters.[4–7] For example, levels of miR-34-5p together with YKL-40 (chitinase-3-like protein 1) are monitored in the NIS2+™ panel to monitor MASLD progression.[4]

In this study, we analyzed the serum levels of a 10-miRNA panel in 70 MASLD patients, 47 HCC patients and 14 healthy controls. All individuals were genotyped for the *PNPLA3* c.444 C>G rs738409 polymorphism. The risk allele encodes the I148M variant of the PNPLA3 lipase which facilitates hepatic steatosis and increases the risk for liver diseases, including MASLD and HCC. The variant allele has effects on the transcriptome of liver cells.[8,9] Of note, few studies also reported about distinct miRNA profiles in individuals with chronic liver diseases carrying the *PNPLA3* p.I148M risk allele.[10–12] Here we demonstrate that miR-335-5p performs very well as a predictor of HCC in receiver operating characteristic (ROC) curve analyses. Moreover, we describe miR-4651 as a novel predictor for MASLD and for MASLD complication. We did not observe an impact of the *PNPLA3* genotype on the serum levels of the miRNAs.

Patients and Methods

Patient Samples

Serum samples from healthy individuals and from patients with chronic liver disorders (MASLD or HCC) were collected at the Saarland University Hospital in Homburg (Germany) at the Institute for Occupational and Environmental Medicine and Public Health or the Department of Medicine II, respectively. Liver-specific diseases were excluded in the healthy controls, and they did not present with elevated liver function tests. The presence of chronic liver diseases other than MASLD—particularly alternative causes of hepatic steatosis such as alcohol-associated liver disease (threshold of alcohol consumption 20 g/day in women and 30 g/day in men) or drug-induced liver injury—was excluded in all patients; none of the patients showed signs of autoimmune, cholestatic, or viral liver diseases. HCC was diagnosed either histologically or non-invasively using contrast-enhanced imaging according to the Liver Imaging Reporting and Data System computer tomography/magnetic resonance imaging v2018 criteria.[13]

After collection, the whole blood samples were kept at room temperature for 30 minutes for clotting. Then, the tubes were centrifuged (2000 g, 10 minutes, 4 °C). Serum samples were kept at −80 °C until miRNA extraction. Genomic DNA was isolated from ethylenediaminetetraacetic acid-anticoagulated blood samples using the membrane-based QIAamp DNA extraction protocol (Qiagen). The rs738409 polymorphism in *PNPLA3* was assessed by polymerase chain reaction (PCR) (TaqMan, assay ID C_7241_10). For the results shown in this manuscript, we compared the homozygote wild-type (WT) genotype [CC] with the "variant" genotypes ([CG] + [GG]). For 101 of the 131 sera, we had previously quantified the levels of 22 cytokines, chemokines, growth factors, or soluble receptors (commonly referred to as "cytokines").[14] Liver stiffness measurements by transient elastography (Fibroscan) are available for most MASLD patients and the healthy individuals. Tables 1 and A1 provide further information on the cohort.

MiRNA extraction from serum samples, quality control

We based the protocol largely on our previous study.[15] For the HCC samples, we extracted miRNA as duplicates from 200 μL of serum each. We mixed the 2 duplicates, if both fulfilled the quality criteria (see below). For the MASLD samples, due to smaller volumes of serum available, we extracted miRNA as unicates from 50 μL. For the healthy samples, we extracted miRNA twice (in parallel to the patient samples, to ensure the same technical conditions), once as duplicates (200 μL) and once as unicates (50 μL); we analyzed the 2 batches separately in downstream analyses.

Table 1. Characteristics of Patients Included in the Cohort (N = 131)

Parameters	Healthy (N = 14)	MASLD-NC-Stea (simple steatosis) (N = 40)	"MASLD complication" (N = 30): MASLD-NC-MASH (no cirrhosis, MASH) (N = 9)	"MASLD complication" (N = 30): MASLD-Cirr (cirrhosis) (N = 21)	HCC-NC (no cirrhosis) (N = 15)	HCC-Cirr[a] (cirrhosis) (N = 32)	Overall (N = 131)
PNPLA3 p.I148M genotype							
CC	9 (64.3%)	20 (50.0%)	4 (44.4%)	10 (47.6%)	3 (20.0%)	10 (31.3%)	56 (42.7%)
CG	3 (21.4%)	17 (42.5%)	2 (22.2%)	10 (47.6%)	8 (53.3%)	12 (37.5%)	52 (39.7%)
GG	2 (14.3%)	3 (7.5%)	3 (33.3%)	1 (4.8%)	4 (26.7%)	10 (31.3%)	23 (17.6%)
Gender							
F	8 (57.1%)	16 (40.0%)	5 (55.6%)	12 (57.1%)	2 (13.3%)	10 (31.3%)	53 (40.5%)
M	6 (42.9%)	24 (60.0%)	4 (44.4%)	9 (42.9%)	13 (86.7%)	22 (68.8%)	78 (59.5%)
Age							
Mean (SD)	49.6 (9.52)	55.9 (12.9)	47.1 (10.5)	59.7 (15.5)	70.9 (6.57)	66.6 (10.8)	59.6 (13.7)
Median [min, max]	49.5 [25.0,67.0]	55.5 [23.0, 80.0]	48.0 [31.0, 59.0]	62.0 [31.0, 84.0]	72.0 [58.0, 80.0]	64.0 [45.0, 92.0]	61.0 [23.0, 92.0]
BMI							
Mean (SD)	30.2 (4.25)	28.5 (4.81)	29.1 (7.66)	25.3 (6.11)	29.0 (4.48)	29.7 (6.50)	28.8 (5.66)
Median [min, max]	29.5 [25.3, 38.3]	28.6 [17.6, 41.1]	26.0 [21.8, 41.0]	24.8 [17.3, 37.3]	29.3 [21.2, 38.1]	28.7 [19.9, 46.2]	28.3 [17.3, 46.2]
Missing	0 (0%)	15 (37.5%)	4 (44.4%)	7 (33.3%)	0 (0%)	0 (0%)	26 (19.8%)
Fibroscan (kPa)							
Mean (SD)	5.27 (1.53)	5.34 (1.58)	7.06 (2.00)	37.3 (26.1)	/	/	12.3 (17.6)
Median [min, max]	4.55 [3.60, 8.70]	5.00 [2.20, 9.10]	6.30 [4.00, 10.2]	26.3 [10.1, 75.0]	/	/	5.60 [2.20, 75.0]
Missing	0 (0%)	0 (0%)	0 (0%)	4 (19.0%)			4 (4.8%[b])

Demographics, *PNPLA3* p.I148M genotype, BMI, and "Fibroscan" (liver stiffness assessed by transient elastography) are indicated for the 6 groups: Healthy, MASLD-NC-Stea, MASLD-NC-MASH, MASLD-Cirr, HCC-NC, HCC-Cirr. Age, BMI, and Fibroscan are presented as mean ± SD, and median. Moreover, the numbers of missing values are indicated.

BMI, body mass index; SD, standard deviation.

[a]Of the 32 patients, 22 were classified as Child A, 8 as Child B, and 2 as Child C.

[b]For Fibroscan, no data were collected for HCC patients. Thus, the percentage was calculated only for the healthy and MASLD samples (N = 84).

Before the addition of Qiazol, the serum was thawed at room temperature and centrifuged (30 minutes, 16,000 g, 4 °C) to remove cellular debris. The miRNA extraction was performed using the miRNeasy serum/plasma kit from Qiagen. We incorporated 3 exogenous *C. elegans* miRNAs as spike-in controls, cel-miR-39-3p, cel-miR-54-3p, and cel-miR-238-3p, at different concentrations to account for potential biases in the quantification of miRNAs with different levels of abundance. Furthermore, cel-miR-39-3p was used for standardization of data across all samples (see below).

Real-time quantitative polymerase chain reaction quality control was performed prior to array analysis using primers for cel-miR-39-3p, cel-miR-54-3p and cel-miR-238-3p, to control for variations in recovery and amplification efficiency between RNA preparations. Additionally, we assessed levels of miR-451a (highly expressed in erythrocytes) and of miR-23a-5p (high levels in serum and not in blood cells) as an indication for possible hemolysis and of SNORD95 to control for potential white blood cell contamination. 4 μL (for 200 μL extractions) or 6 μL (for 50 μL extractions) out of 14 μL eluted total RNA were reverse-transcribed in a 10 μL reaction volume with the miScript RT II kit (Qiagen) following the supplied protocol using Hispec buffer, which selectively amplifies only mature miRNAs. Real-time PCR detection of the above-mentioned mature miRNAs and of SNORD95 was carried out on a CFX96 Detection System (Bio-Rad) using 1 μL of 1:10 diluted cDNA, 2x iQ SYBR Green Supermix (BioRad), and 10$\times$ miRNA-specific primer assay (Qiagen). Specificity of the qPCR primers was assessed by a post-qPCR melting curve analysis. Samples not reaching sufficient quality metrics due to hemolysis, white blood cell contamination, or incomplete recovery of spiked-in controls were discarded.

MiRNA Profiling by qPCR Arrays, Quality Control and Data Analysis

Ten miRNAs were selected for analysis with customized qPCR arrays (Qiagen): miR-122-5p, miR-146a-5p, miR-146b-5p, miR-21-5p, miR-335-5p, miR-433-3p, miR-4530, miR-4651, miR-526a2, and miR-873-5p. Before proceeding to the qPCR arrays, due to generally low amounts of miRNAs in serum samples, we implemented a preamplification step with 1:5 diluted cDNA using the miScript PreAMP PCR kit (Qiagen) along with the appropriate primer mixes (custom primer mix for custom qPCR arrays). Preamplification control experiments were performed by real-time quantitative polymerase chain reaction with primer assays targeting SNORD95, cel-miR-39-3p, and miRTC (internal miRNA reverse transcription control). miR-335-5p, miR-21-5p, and miR-122-5p were detected at highest levels (mean Cq values of 15.4, 16.5, and 17.5), miR-433-3p, miR-4651, and miR-146a-5p at intermediate levels (mean Cq values: 19.2, 19.6, and 20.6), while miR-146b-5p, miR-4530, miR-873-5p, and miR-526a were detected with the lowest levels (mean Cq values: 23.4, 23.8, 25.8, and 26.7). Of note, the relative values for miR-21-5p, miR-122-5p, and miR-146b-5p which we obtained upon preamplification match well to the ones obtained by López-Riera *et al.*, without amplification (Cq values of 28, 29, and 37, respectively[5]). MiRNAs were profiled using custom qPCR arrays (Qiagen) and a CFX384 Detection System (Bio-Rad). Each qPCR array plate included default internal controls (miRTC, positive PCR control, and cel-miR-39-3p).

For qPCR array analysis, baselines and thresholds were adjusted according to the supplier's recommendations and Cq values were extracted for further analysis. Cq values $>$ 40 or samples exhibiting bad melting curves were considered as "not detected". To assess the quality of the melting curves, we used a Support Vector Machine–based method generated with the R 'e1071' package.[15] Cq values obtained with the cel-miR-39-3p primers were used to calibrate the data sets: The mean Cq value of the two technical duplicates for cel-miR-39-3p was subtracted from each sample's Cq value. From this calibrated data, we calculated the sample expression using the formula: "2ˆ-cq_calibrated".

qPCR Analysis of MiRNAs Isolated from Primary Human Liver Cells upon Stimulation with Hyper-Interleukin-6

See supporting information, p. 2.

Statistical Analysis

Differences between groups in levels of each of the 10 miRNAs were tested, separately for the "50 μL" and "200 μL" batches, using pairwise t-tests from the rstatix package,[16] followed by family-wise Holm adjustment. If only 2 groups were tested, a Benjamini-Hochberg (BH) correction[17] was applied between variables. Associations between categorical factors were assessed using a chi-square test. Values for all miRNAs, clinical parameters, and cytokines were log-transformed (for exceptions see[14]). Correlations between miRNAs and cytokines or numeric clinical parameters (with at least 10 pairs of values, see[14] for further information) were analyzed with Pearson correlation and P values were corrected using the BH algorithm. The basis of the predictions was a generalized linear model model using a binomial distribution. This model predicts the binary class of a dependent variable (y), given one or several independent variable(s). It was run for each of the 10 miRNAs; P values were corrected using the BH algorithm. When this generalized linear model showed an adjusted P value $<$.05, we selected it for further ROC curve analysis. The cut-off represents the threshold in y value (class prediction) and was chosen as giving the highest Youden's index. For models with BH-adjusted P values $<$0.05, we also built models with more variables (second miRNA, a cytokine, clinical parameter, or genotype), with the maximum of 1 predictor per 10 events ("rule-of-thumb limit"). When several variables were included in a model, we excluded correlated pairs of variables (ie, $P < .05$, $R > 0.4$ or $R < -0.4$) to avoid collinearity. All data analysis was conducted using R version 4.2.2[18] and rstatix package.[16] Graphs were generated using ggplot2[19] and corrplot.[20] ROC curves were obtained using ROCit package.[21]

Results

Study Cohort and PNPLA3 *p.I148M* Genotype Distribution

We quantified the levels of miRNAs in sera of patients with MASLD or HCC: 70 individuals with MASLD (21 with cirrhosis, "MASLD-Cirr"; 9 with MASH but without cirrhosis, "MASLD-NC-MASH"; 40 with simple steatosis, "MASLD-NC-

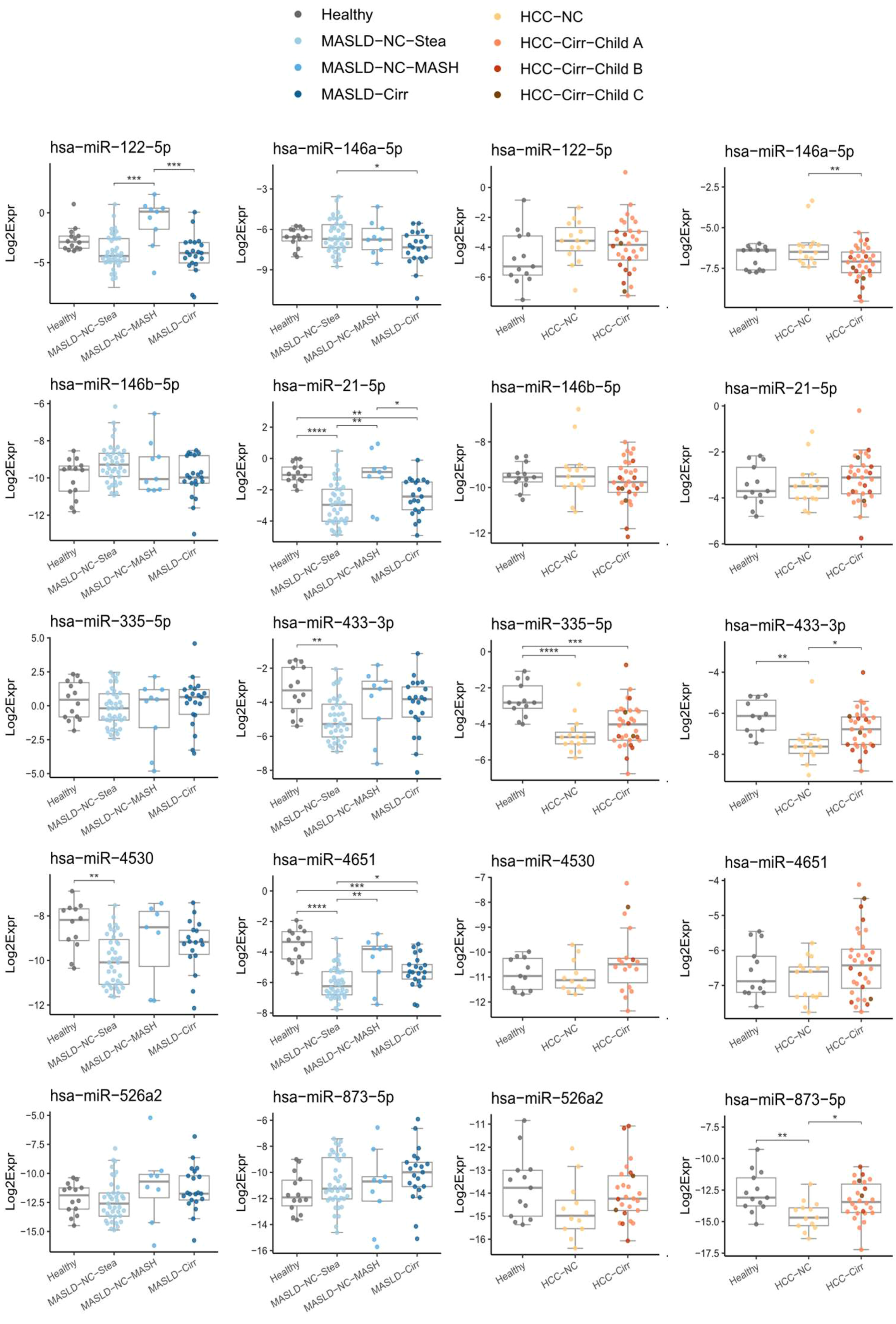

Figure 1. Profiles of 10 miRNAs in patients with chronic liver diseases. The horizontal line within the box plots represents the median, vertical lines from the boxes (whiskers) indicate the variability outside the upper and lower quartiles. Pairwise t-tests were performed, and *P* values were adjusted for each miRNA using the Holm method. ****: $P < .0001$, ***: $P < .001$, **: $P < .01$, *: $P < .05$.

Stea"), 47 individuals with HCC (32 with cirrhosis, "HCC-Cirr"; 15 without, "HCC-NC"). 14 healthy individuals without chronic liver diseases were in the control group. Demographics and some characteristics of the cases and controls are presented in Table 1; more information on clinical parameters is available in Table A1. All individuals were genotyped for their allele status of *PNPLA3*. In total, 42.7% of the participants were homozygous for the WT allele [CC], 39.7% were heterozygous [CG], and 17.6% were homozygous for the variant allele [GG].

Focus on 10 Circulating MiRNAs with Possible Relevance to Inflammatory Liver Diseases

For this study, we selected a panel of 10 miRNAs: MiR-146b-5p, miR-4530, miR-4651 were chosen as we identified them to be upregulated by the inflammatory cytokine hyper-interleukin-6 (IL-6) in primary hepatocytes[22] and (Figure A1); miR-146a-5p was included for comparison with miR-146b-5p. MiR-526a2 (equivalent to miR-518d-5p and miR-520c-5p) and miR-873-5p were included based on previous evidence suggesting their relevance in liver diseases.[23,24] Based on preliminary unpublished data, we added miR-335-5p and miR-433-3p, which were well detectable in the supernatant of hepatocytes (Cq values $<$ 30) and at increased concentration upon hyper-IL-6-stimulation. Our miRNA panel also included miR-122-5p and miR-21-5p, both well-studied miRNAs in the context of inflammation and liver diseases, (eg[5,25–27]).

Altered MiRNA Profiles in Sera from Patients with Chronic Liver Diseases

The serum levels of miR-335-5p, miR-433-3p, and miR-873-5p were decreased in HCC patients compared to healthy controls (Figure A2). When analyzing HCC subgroups, categorized according to the presence or absence of cirrhosis, circulating levels of miR-433-3p and miR-873-5p were higher in HCC patients with cirrhosis (HCC-Cirr) compared to those without (HCC-NC). In contrast, serum concentrations of miR-146a-5p were lower in HCC patients with cirrhosis (Figure 1).

In the MASLD patients, serum levels of miR-21-5p, miR-433-3p, miR-4530, and miR-4651 were decreased compared to healthy controls (Figure A2). The decrease of miR-4530 and miR-433-3p levels in the sera from MASLD patients (compared to the healthy controls) was significant only for patients with simple steatosis (MASLD-NC-Stea; Figure 1). Sera of patients with MASH (MASLD-NC-MASH) showed higher levels of miR-122-5p and of miR-21-5p as compared to patients with simple steatosis (MASLD-NC-Stea) and to patients with MASLD and cirrhosis (MASLD-Cirr). For miR-4651, both groups with complicated MASLD (MASH, cirrhosis) displayed higher serum levels than the simple steatosis group (Figure A3A). Similar findings (albeit with higher *P* values) were observed for miR-21-5p, miR-433-3p, miR-4530, and miR-526a2, while miR146a-5p and miR-146b-5p were lower in patients with complicated MASLD (Figure A3A). Notably, miR-146a-5p levels were significantly lower in patients with MASLD-cirrhosis compared to those with simple steatosis, similar to the observation for HCC patients, with or without cirrhosis (see above, Figure 1).

MiRNAs as Predictive Factors for Liver Diseases

We evaluated the suitability of the miRNAs in our panel to serve as discriminative markers in ROC curve analysis-based prediction models.

MiR-335-5p Discriminates Best HCC from Healthy Samples

MiR-335-5p, found at lower levels in HCC as compared to healthy samples (Figures 1, A2), was the only miRNA showing

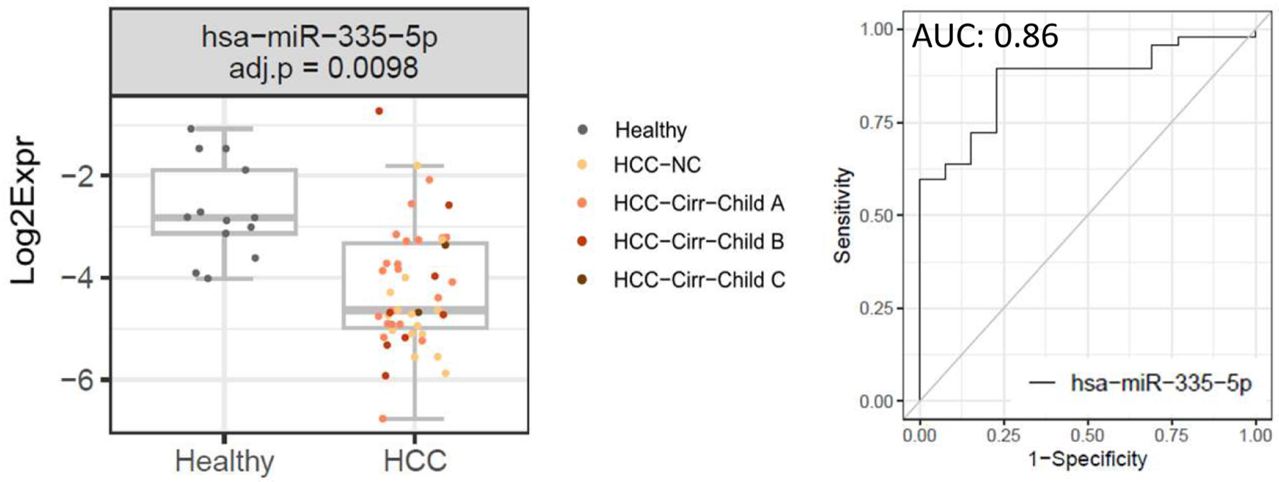

Figure 2. MiR-335-5p as predictor of HCC *vs* healthy controls. The ROC curve for miR-335-5p is shown as well as the concentration plots for the healthy group (14 samples) and the HCC group (47 samples), indicating also the BH-adjusted *P* value of the GLM. GLM, generalized linear model.

Table 2. ROC Curve Analyses

x (/x2)	AUC	Cut-off	PPV	NPV	SENS	SPEC	Equation
Prediction: HCC *vs* healthy (Figure 2)							
AST	0.96	0.65	0.94	0.85	0.94	0.85	y ~ −26.29 + 19.01 *x
GGT	0.96	0.85	0.97	0.65	0.84	0.93	y ~ −9.65 + 6.40 *x
PNPLA3 p.I148M “variant” ([CG] + [GG])	0.68	0.87	0.87	0.41	0.72	0.64	y ~ 0.37 + 1.55 *x
miR-335-5p	0.86	0.71	0.93	0.67	0.89	0.77	y ~ −2.73 + −1.15 *x
Prediction: MASLD-NC stea *vs* healthy (Figure 3A)							
miR-4651	0.95	0.73	0.97	0.72	0.88	0.93	y ~ −8.49 + −1.98 *x
miR-21-5p	0.89	0.74	0.97	0.62	0.79	0.93	y ~ −1.74 + −1.53 *x
miR-4530	0.82	0.56	0.88	0.78	0.95	0.58	y ~ −9.78 + −1.19 *x
miR-433-3p	0.82	0.67	0.87	0.60	0.85	0.64	y ~ −2.71 + −0.91 *x
miR-122-5p	0.75	0.79	1.00	0.48	0.62	1.00	y ~ −0.58 + −0.48 *x
miR-146b-5p	0.68	0.83	0.94	0.35	0.38	0.93	y ~ 8.65 + 0.79 *x
Prediction: MASLD *vs* healthy (Figure 3B)							
GGT	0.94	0.79	0.95	0.76	0.84	0.93	y ~ −9.52 + 6.18 *x
AST	0.93	0.79	0.95	0.75	0.84	0.92	y ~ −21.31 + 15.49 *x
miR-4651	0.90	0.88	0.98	0.45	0.77	0.93	y ~ −4.92 + −1.44 *x
miR-21-5p	0.84	0.89	1.00	0.34	0.61	1.00	y ~ −0.06 + −0.97 *x
Prediction: MASLD-comp *vs* MASLD-NC-stea (Figures 4, A3B)							
miR-4651	0.72	0.39	0.64	0.79	0.77	0.68	y ~ 3.75 + 0.72 *x
miR-4651/Fibroscan	0.95	0.33	0.89	0.95	0.92	0.92	y ~ 13.49 + 1.57 *x + −0.01 *x2
miR-4651/Thrombocytes	0.89	0.43	0.85	0.91	0.88	0.89	y ~ −5.96 + 0.85 *x + 11.72 *x2
miR-4651/GROα	0.84	0.53	0.79	0.83	0.76	0.85	y ~ 28.08 + 0.67 *x + −10.62 *x2
miR-4651/HGF	0.82	0.53	0.86	0.77	0.62	0.92	y ~ 6.37 + 0.78 *x + 2.27 *x2
miR-4651/miR-146a-5p	0.81	0.57	0.86	0.77	0.63	0.92	y ~ 1.31 + 0.80 *x + 2.71 *x2
miR-4651/SCGFβ	0.79	0.46	0.72	0.84	0.79	0.78	y ~ −5.34 + 0.71 *x + 4.80 *x2
miR-4651/CTACK	0.78	0.37	0.68	0.83	0.79	0.72	y ~ −12.42 + 0.66 *x + 6.00 *x2
miR-4651/miR-146b-5p	0.78	0.63	0.94	0.75	0.57	0.97	y ~ 8.95 + 0.93 *x + −1.10 *x2
miR-4651/IL-8	0.76	0.37	0.67	0.85	0.83	0.70	y ~ −0.06 + 1.20 *x + −0.96 *x2
Fibroscan	0.92	0.40	0.88	0.88	0.81	0.92	y ~ −9.53 + 10.44 *x
Thrombocytes	0.87	0.42	0.88	0.89	0.85	0.91	y ~ 25.6 + −11.18 *x
GROα	0.81	0.54	0.9	0.79	0.66	0.95	y ~ −9.66 + 5.00 *x
HGF	0.77	0.60	0.94	0.75	0.55	0.98	y ~ −16.37 + 6.08 *x
miR-146a-5p	0.64	0.36	0.52	0.76	0.83	0.41	y ~ −3.3 + −0.45 *x
SCGFβ	0.71	0.35	0.6	0.85	0.86	0.58	y ~ −13.86 + 3.14 *x
CTACK	0.72	0.37	0.58	0.77	0.76	0.60	y ~ −6.91 + 2.74 *x
miR-146b-5p	0.64	0.46	0.62	0.73	0.67	0.69	y ~ −4.78 + −0.47 *x
IL-8	0.66	0.41	0.64	0.7	0.55	0.78	y ~ −5.79 + 3.90 *x

For the ROC curve analyses shown in the indicated figures, the AUC values, positive predictive values, negative predictive values, and the values for sensitivity (SENS) and specificity (SPEC) are indicated. The cut-offs refer to the outcome of the regression (y). x, x2: independent parameters; y: binary outcome (0/1).
SENS, sensitivity; SPEC, specificity; NPV, negative predictive value; PPV, positive predictive value; GROα, growth-regulated oncogene-alpha; HGF, hepatocyte growth factor; SCGFβ, stem cell growth factor-beta.

statistical significance to discriminate HCC cases from healthy individuals, with an area under the curve (AUC) value of 0.86 (Figure 2) and high scores for sensitivity and specificity (Table 2). However, it is to note that miR-335-5p does not outperform routine clinical parameters for liver damage, ie, gamma-glutamyl transferase (GGT) and aspartate amino transferase (AST), which were both detected at higher levels in sera from HCC patients in comparison to healthy controls (AUC values of 0.96; Table 2).

ROC Curve Analyses Identify MiR-4651 as a Potent Novel Predictor of MASLD

For six miRNAs, the prediction analyses to discriminate healthy from MASLD-NC-Stea (ie, simple steatosis) samples yielded significant results. MiR-4651 performed best, with an AUC value of 0.95 and high values for the positive and negative predictive values, sensitivity, and specificity (Figure 3A, Table 2). MiR-21-5p, miR-4530, and miR-433-3p follow, with AUC values of 0.89, 0.82, and 0.82, respectively. Notably, thrombocytes, as the clinical parameter performing best in this analysis, only yielded an AUC of 0.71 (data not shown).

MiR-4651 also performed best in the ROC curve analyses for discrimination of (all) MASLD from healthy samples (AUC: 0.90), followed by miR-21-5p, miR-4530, and miR-433-3p, with AUC values of 0.84, 0.76, and 0.74, respectively (Figure 3B, Table 2). For this comparison, the clinical parameters GGT and AST yielded better prediction results (AUCs: 0.94 and 0.93, respectively; Table 2).

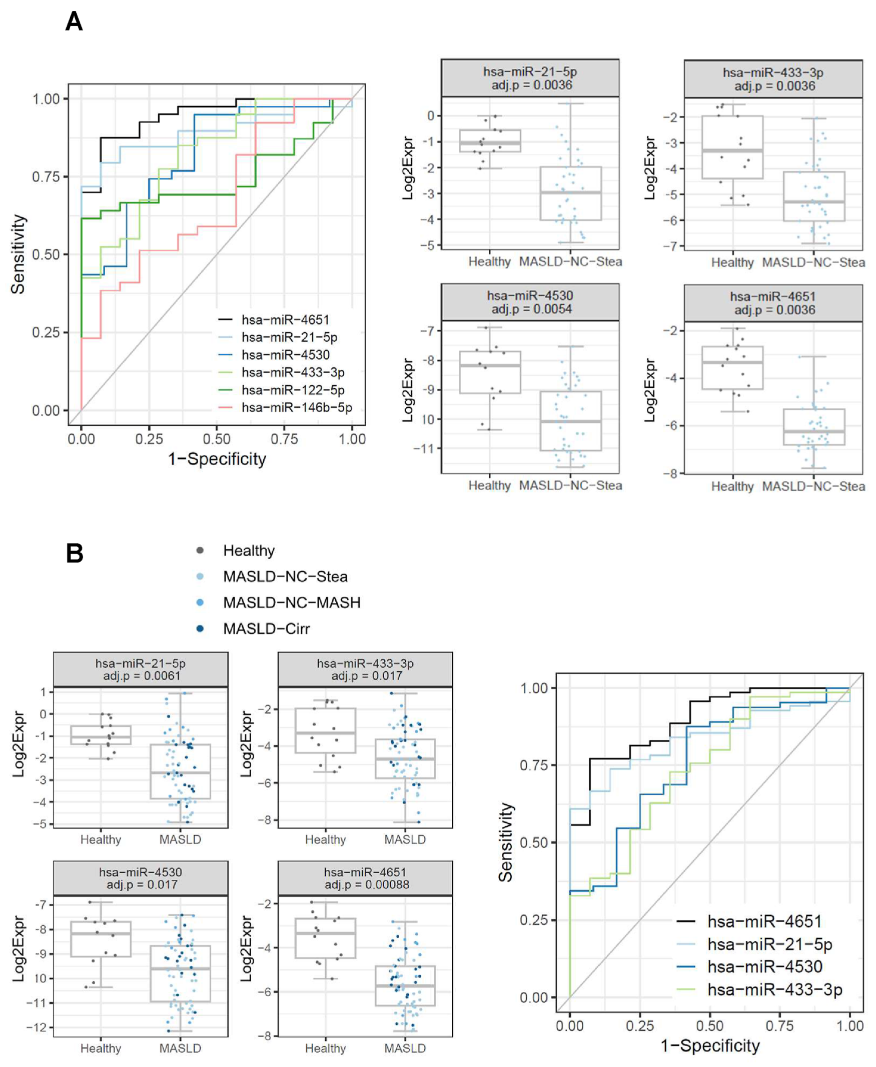

Figure 3. ROC curve analyses for prediction of MASLD *vs* healthy controls. (A) Discrimination of simple steatosis from healthy samples. Left: ROC curves are shown for 6 miRNAs: miR-4651, miR-21-5p, miR-4530, miR-433-3p, miR-122-5p, and miR-146-5p. Right: Concentration plots for the 4 best performing miRNAs, for the healthy group (14 samples) and the MASLD-NC-Stea group (40 samples). The BH-adjusted *P* values of the GLMs are indicated. (B) miR-4651 and miR-21-5p are best predictors to differentiate (all) MASLD from healthy samples. Left: Plots of the relative levels of the 4 miRNAs with BH-adjusted *P* values of the GLMs $<$0.05: miR-4651, miR-21-5p, miR-4530, and miR-433-3p; the healthy group comprises 14 samples, the MASLD group 70 samples. Right: ROC curves for these 4 miRNAs. GLM, generalized linear model.

Interestingly, miR-4651 was the only miRNA predicting, within the MASLD group, “complicated” MASLD cases (MASLD-Comp) *vs* uncomplicated ones (MASLD-NC-Stea, ie, simple steatosis); Figure 4). The moderate AUC value of 0.72 could be improved by adding a second parameter into the model: including miR-146a-5p or miR-146b-5p into the ROC curve analyses led to increased AUC values of 0.81 and 0.78, respectively. Adding serum levels of inflammatory cytokines such as growth-regulated oncogene-alpha, hepatocyte growth factor, stem cell growth factor-beta, or CTACK increased the AUCs to reach values of 0.84, 0.82, 0.79, and 0.78, respectively. (As shown in our previous manuscript[14] and in Table 2, growth-regulated oncogene-alpha was most effective among 22 cytokines in a single-parameter model to predict complicated MASLD (AUC: 0.78), followed by hepatocyte growth factor, stem cell growth factor-beta, and CTACK (Table 2). As expected, the combination of miR-4651 with thrombocytes or liver stiffness (quantified by transient elastography) led to very high AUC values of 0.95 and 0.89, respectively (Figures 4; A3B and Table 2 also present their excellent values in single-parameter models).

Effect of the PNPLA3 *p.I148M Risk Allele on Levels of Circulating MiRNAs*

We analyzed whether the *PNPLA3* p.I148M genotype was associated with the levels of the serum miRNAs. We noted lower levels of miR-21-5p in carriers of the risk allele in the steatosis group, whereas for the other comparisons no differences were detected (Figure 5 and data not shown), although miR-122-5p has been previously reported to be present at higher concentrations in carriers of the risk allele.[10] Of note, in all groups miR-4530 levels tended to be decreased in carriers of the risk allele (Figure 5).

Discussion

In this study we show that 1) miR-4651 is the best predictor for MASLD. In particular, this miRNA can differentiate individuals with simple steatosis from healthy ones, it also emerged as the best predictor for “MASLD complication” *vs* simple steatosis; 2) miR-335-5p is present at lower levels in patients with HCC compared to healthy

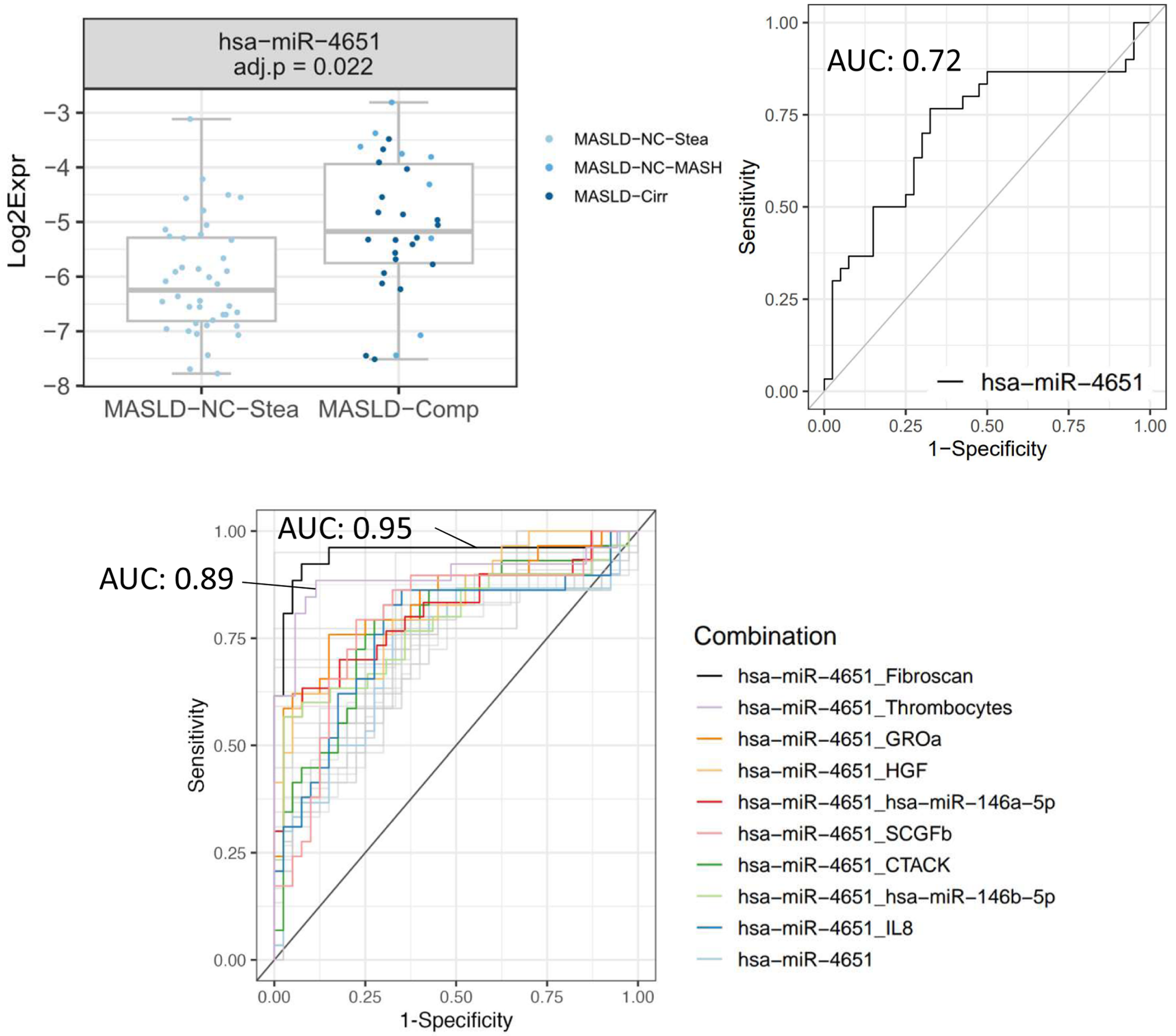

Figure 4. Two-parameter models including miR-4651 for an improved discrimination of "MASLD complication" from simple steatosis. Top left: Plots of the relative levels of miR-4651 in the simple steatosis group (MASLD-NC-Stea, 40 samples) and in the "MASLD complication" group (MASLD-Comp: 30 samples: 9 for MASLD-NC-MASH, 21 for MASLD-Cirr); the BH-adjusted *P* value of the GLM is indicated. Top right: ROC curve for miR-4651. Lower panel: The addition of second parameters to the model leads to increased AUC values. Among the significant combinations, the 10 best AUC combinations are colored and labeled, the others are shown in gray. GLM, generalized linear model.

controls; and 3) the *PNPLA3* p.I148M risk genotype does not seem to strongly affect serum levels of the 10 miRNAs.

MiR-4651 as a Novel MiRNA with Biomarker Potential for MASLD

MiR-4651 is downregulated in the sera of MASLD patients compared to healthy controls whereas no significant difference was observed between the HCC and healthy samples (Figures 1, A2). Of note, levels are particularly low for the MASLD-NC-Stea (ie, simple steatosis) group, with higher levels for the MASLD-NC-MASH and MASLD-Cirr groups. In ROC curve analyses, miR-4651 performed best in discriminating 1) healthy samples from MASLD-NC-Stea samples (Figure 3A), 2) healthy samples from (all) MASLD samples (Figure 3B), and 3) samples from MASLD patients with simple steatosis from those with complication (i.e., MASH/cirrhosis) (Figure 4). Of note, miR-4651 performed better than the "best" clinical parameter (thrombocytes) to predict simple steatosis *vs* healthy controls. In contrasts, for other prediction models (healthy *vs* MASLD, simple steatosis *vs* MASLD complication) the clinical parameters GGT and AST or "Fibroscan" and "thrombocytes" were more powerful than any of the miRNAs (Table A1, Figure A3B). In fact, combinations of miR-4651 with the parameters "Fibroscan" or "thrombocytes" led to the highest AUC levels in the model differentiating MASLD without complication (ie, simple steatosis) from MASLD with complication.

Only a few studies have reported on this miRNA so far. MiR-4651 was one of well-detectable serum miRNAs in a 8-

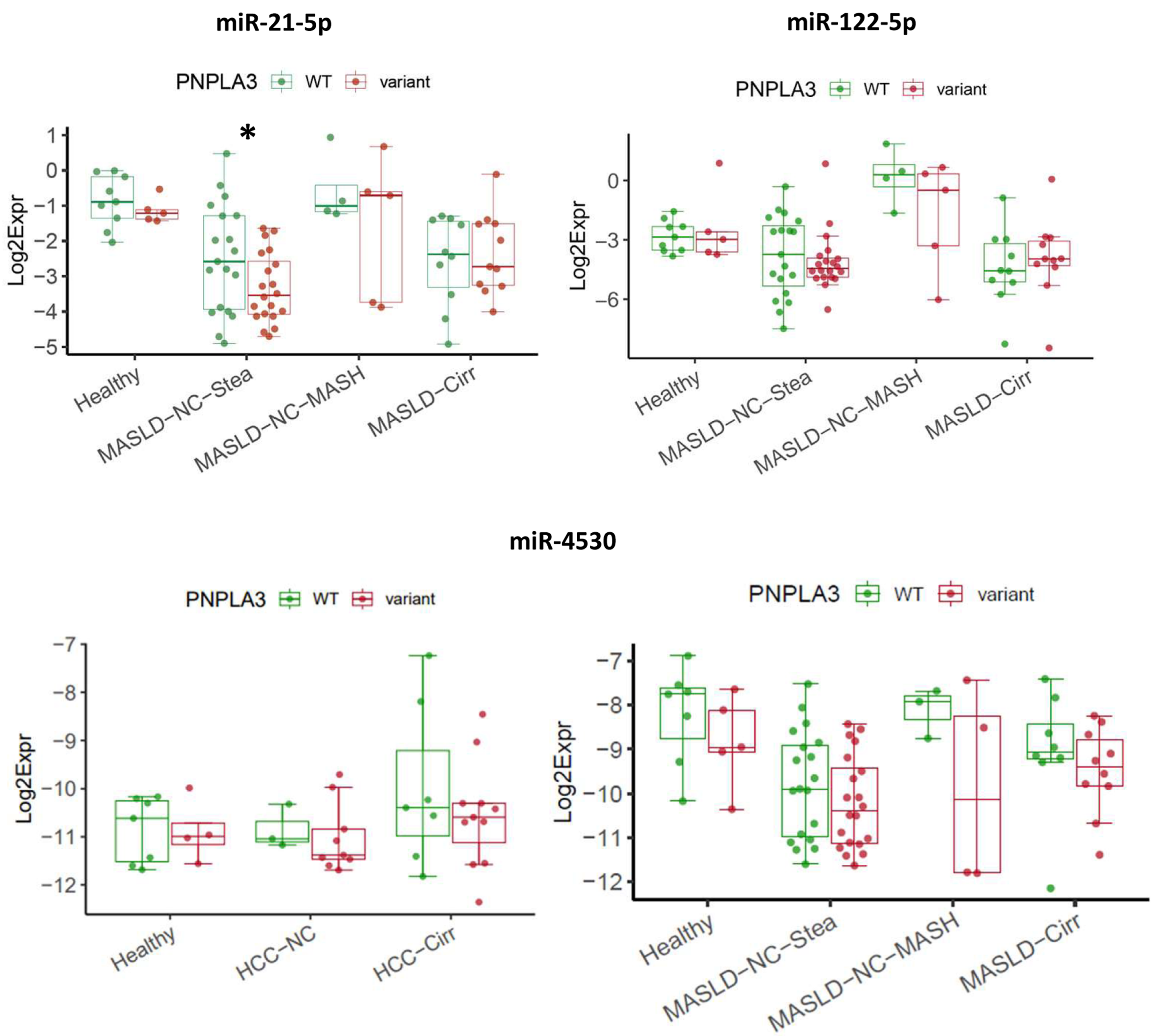

Figure 5. The *PNPLA3* p.I148M risk allele does not substantially affect levels of circulating miRNAs. Upper panel: Lower levels of miR-21-5p in carriers of the variant allele ([CG] + [GG]) compared to the homozygous carriers of the WT allele [CC] in the steatosis group, *: $P = .046$. Lower panel: Tendency (but no statistical significance) of lower levels of miR-4530 in carriers of the variant allele in all groups.

miRNA panel able to predict HCC in patients with various liver diseases.[6] In agreement with our data, Yamamoto *et al.* found that miR-4651 levels were reduced in a large cohort of patients with chronic hepatitis/liver cirrhosis compared to healthy controls and to HCC patients. Moreover, miR-4651 was reported to be a biomarker in HCC cases related to aflatoxin; serum levels of miR-4651 were positively related to the concentration of aflatoxin B1-albumin adduct levels.[7] While Sun *et al.* did not detect differences in the sera of patients with cirrhosis compared to healthy controls,[28] miR-4651 was previously found to be upregulated in the plasma of chronically hepatitis B virus-infected patients with fibrosis stages S1 to S4 compared to those with S0.[29] Moreover, miR-4651 was part of a panel of 9 serum miRNAs that could serve as early biomarkers for new-onset type-2 diabetes, with higher levels in patients than in healthy controls.[30] Expression of miR-4651 was found to be decreased in HCC tissues compared to adjacent normal tissues, and patients with higher expression had a better survival rate. It suppresses the growth of HCC by targeting the expression of the transcription factor FOXP4.[31] High tissue levels of miR-4651 were predictive of a better response to transarterial chemoembolization in HCC patients,[32] possibly linked to expression of its target CYP2W1, a member of the cytochrome P450 superfamily. Taken together, follow-up studies are warranted to better define the role of this miRNA in liver diseases and its potential as a biomarker.

MiR-335-5p Levels are Decreased in HCC

Serum levels of miR-335-5p were significantly lower in HCC patients, regardless of cirrhosis status, compared to healthy controls (Figures 1, A2). In a prediction model,

miR-335-5p performed well in discriminating between HCC and healthy controls (AUC: 0.86, Figure 2). In line with our results, Elfert *et al.* reported decreased serum levels of miR-335 in patients with hepatitis C virus (HCV) (compared to normal controls), especially those with HCV-related HCC. Indeed, this miRNA had a high prognostic power in distinguishing HCC patients from nonmalignant HCV patients or healthy controls.[33] Also Cui *et al.* found serum levels of miR-335-5p to be reduced in HCC patients compared to healthy controls and hepatitis patients; higher miR-335 concentrations were associated with a better treatment response to transarterial chemoembolization and with a better prognosis of HCC patients.[34] Decreased levels of this miRNA were observed in extracellular vesicles isolated from sera of patients with severe liver injury-chronic hepatitis B and decompensated cirrhosis.[35]

MiR-335-5p has tumor suppressive functions in several types of cancer, including HCC (see[36] for a review), inhibiting expression of proteins like ROCK1 or MAPK1, implied in the regulation of cellular migration and/or proliferation.[37,38] *MiR-335* is located within an intron of *MEST*, a gene presenting hypermethylation of its promoter in HCC,[39] which may explain the lower expression of miR-335-5p in tumor tissue compared to the surrounding nontumor tissue.[37–39] Overexpression of miR-335-5p or application of miR-335-5p via exosomes decreased proliferation rates of hepatoma cells, both *in vitro* and *in vivo*.[40] Indeed, an exosome-based anticancer drug using miR-335 (next to tumor necrosis factor-related apoptosis inducing ligand) was started to be developed for treatment of HCC.[36]

Results of Other MiRNAs in the Context of Previous Studies

We included into our study miR-122-5p, the most abundant miRNA in the liver, as well as the inflammation-associated miR-21-5p, both well-studied miRNAs in the context of liver diseases and robustly detectable in serum (eg[5]). In HCC samples, we observe only a trend toward higher levels of both miRNAs than in healthy controls (Figure 1). Several studies, however, described significantly higher levels in the blood of HCC patients, of miR-122 (eg[26,27,33]) or/and of miR-21-5p (eg[26,41]), whereas no differences were noted in other studies.[27,42] (For miR-122-5p, we found higher levels in MASLD-NC-MASH samples compared to those from MASLD-NC-Stea and MASLD-Cirr samples. As this miRNA was previously found to be decreased in high grades of fibrosis (eg[5,43]), it was stated that this miRNA could “be postulated as a biomarker for nonalcoholic steatohepatitis, but it does not seem useful as a biomarker for advanced fibrosis”.[5] Higher concentrations of circulating miR-122-5p in MASH samples compared to simple steatosis samples were also reported by others (eg[25,44,45]). However, in contrast to several other (eg[25,44–47]) but not all[48,49] studies, we did not detect significant differences between (all) MASLD and healthy samples in our cohort (Figure A2). Of note, our MASLD cohort is dominated by individuals with simple steatosis (40, ie, 57%, *vs* 9 with MASH and 21 with cirrhosis). Interestingly, Auguet *et al.* (2014) and Pirola *et al.* (2015[44]; validation cohort) found no differences between healthy and simple steatosis, whereas miR-122 levels were significantly higher in sera from patients with steatohepatitis.[44,50]

MASLD-NC-MASH samples contained significantly higher levels also of miR-21-5p compared to the MASLD-NC-Stea and MASLD-Cirr samples (Figure 1). Moreover, we also observed a higher concentration in “MASLD-complication” samples compared to simple steatosis (Figure A3A). Higher serum levels of miR-21-5p in MASH samples compared to simple steatosis have also been noticed by others.[25,51] It should be noted, however, that the overall levels of miR-21-5p were lower in MASLD samples compared to the healthy samples of our cohort (Figure A2). This is in line with a study by Sun et al.[52] whereas other studies noted no difference[46,47] or higher levels in MASLD (eg[25,26]).

MiR-873-5p and miR-526a2 (sequence identity with miR-518d-5p) were included in the study because of their relevance to liver diseases: miR-873-5p is found to be upregulated in liver tissues of MASLD patients, an important target of miR-873-5p being the mitochondrial glycine-N-methyltransferase, thus affecting the methionine cycle and the activity of the electron transport chain.[23] MiR-526a2 was described to be upregulated in the tissues and sera of HCC patients and to exert antiapoptotic effects in HCC cells.[24] While we only see a trend towards higher levels of serum miR-873-5p during MASLD progression (Figures 1, A2, A3A), miR-526a is higher expressed in “complicated MASLD” compared to simple steatosis (Figure A3A). MiR-873-5p is present at lower levels in sera of HCC patients compared to healthy controls while in our study no significant difference was found for miR-526a2 (Figures 1, A2).

In our previous microarray analysis, miR-4530 and miR-4651 were among the miRNAs that were upregulated in primary human hepatocytes upon stimulation with hyper-IL-6,[22] a finding that we could confirm by qPCR validation, including further primary hepatocyte samples (Figure A1). Interestingly, miR-4530 is present at higher concentration in the sera of MASLD patients “with complication” compared to those with simple steatosis (Figure A3A). Overall, we observe lower levels miR-4530 in MASLD patients compared to healthy controls (Figure A2A), which is driven by a lower concentration in patients with simple steatosis (Figure 1). HCC sera did not present different levels of miR-4530 compared to healthy control samples. Of note, in a study with 86 HCC patients, those with a higher serum level of miR-4530 had better survival rates compared to those with lower levels.[53]

MASLD and HCC patients with cirrhosis had reduced levels of serum miR-146a-5p (Figure 1). Interestingly, this microRNA was also observed to be reduced in hepatic fibrosis due to schistosomiasis, with lower serum levels in higher grades of fibrosis.[54] Also Blaya *et al.* found the circulating levels of miR-146a-5p to be lower in patients with cirrhosis compared to healthy controls, with a further decrease in patients with

decompensated cirrhosis or acute-on-chronic liver failure and correlating with patient outcomes.[55]

No Major Impact of the PNPLA3 *p.I148M Risk Genotype on the Serum Levels of the 10 MiRNAs*

The *PNPLA3* p.I148M allele is one of the strongest genetic risk factors for MASLD and for other liver diseases. Interestingly, we did not observe significant differences between carriers of the risk allele and those with homozygosity of the WT allele (exception: lower levels of miR-21-5p in the sera of MASLD-NC-Stea patients with the risk allele, Figure 5). Our study therefore does not confirm findings by Ezaz *et al.*,[10] who found the GC and GG variants at rs738409 of *PNPLA3* to be robustly associated with higher levels of circulating miR-122-5p in a cohort of 132 MASLD patients. Of note, carriers of the *PNPLA3* p.I148M risk allele had consistently lower median values regarding the serum level of miR-4530 compared to those carrying only the WT allele (Figure 5).

Conclusion

It would be important to validate our findings in an independent study cohort with a larger number of enrolled individuals, including a larger healthy control group (ours comprised 14 participants only), to increase statistical power. Moreover, the suitability of miR-4651 as biomarker in the diagnosis of simple steatosis should be followed up. A limited number of molecules have been tested for their diagnostic potential to detect simple steatosis: Soluyanova *et al.* identified a set of 5 serum miRNAs which could predict liver fat content with high sensitivity and specificity.[56] Moreover, serum concentrations of the liver-produced glycoprotein afamin correlate with hepatic fat contents and could therefore serve as an early marker of metabolic syndrome.[57] For prediction of MASH and MASLD-cirrhosis, more powerful clinical markers, as single parameters, outperformed the miRNAs of our panel. Yet, we provide evidence that (selected) miRNAs may be valuable analytes for inclusion in multiparameter prediction models, to complement imaging techniques and serum markers as well-established noninvasive diagnostic tools for MASLD.[1,58]

Supplementary Materials

Material associated with this article can be found, in the online version, at https://doi.org/10.1016/j.gastha.2025.100839.

References

1. Huang DQ, Wong VWS, Rinella ME, et al. Metabolic dysfunction-associated steatotic liver disease in adults. Nat Rev Dis Primers 2025;11:14.
2. Singal AG, Kanwal F, Llovet JM. Global trends in hepatocellular carcinoma epidemiology: implications for screening, prevention and therapy. Nat Rev Clin Oncol 2023;20:864–884.
3. Llovet JM, Kelley RK, Villanueva A, et al. Hepatocellular carcinoma. Nat Rev Dis Primers 2021;7:6.
4. Harrison SA, Ratziu V, Magnanensi J, et al. NIS2+, an optimisation of the blood-based biomarker NIS4(R) technology for the detection of at-risk NASH: a prospective derivation and validation study. J Hepatol 2023;79:758–767.
5. Lopez-Riera M, Conde I, Tolosa L, et al. New microRNA biomarkers for drug-induced steatosis and their potential to predict the contribution of drugs to non-alcoholic fatty liver disease. Front Pharmacol 2017;8:3.
6. Yamamoto Y, Kondo S, Matsuzaki J, et al. Highly sensitive circulating MicroRNA panel for accurate detection of hepatocellular carcinoma in patients with liver disease. Hepatol Commun 2020;4:284–297.
7. Wu XM, Xi ZF, Liao P, et al. Diagnostic and prognostic potential of serum microRNA-4651 for patients with hepatocellular carcinoma related to aflatoxin B1. Oncotarget 2017;8:81235–81249.
8. Baselli GA, Dongiovanni P, Rametta R, et al. Liver transcriptomics highlights interleukin-32 as novel NAFLD-Related cytokine and candidate biomarker. Gut 2020;69:1855–1866.
9. Caon E, Martins M, Hodgetts H, et al. Exploring the impact of the PNPLA3 I148M variant on primary human hepatic stellate cells using 3D extracellular matrix models. J Hepatol 2024;80:941–956.
10. Ezaz G, Trivedi HD, Connelly MA, et al. Differential associations of circulating MicroRNAs with pathogenic factors in NAFLD. Hepatol Commun 2020;4:670–680.
11. Youssef SS, Youness RA, Abbas EAE, et al. miR-516a-3P, a potential circulating biomarker in hepatocellular carcinoma, correlated with rs738409 polymorphism in PNPLA3. Per Med 2022;19:483–493.
12. Donati B, Vaira V, Donnangelo A, et al. A distinct miRNA expression profile in hepatocellular carcinoma arising in carriers of the I148M PNPLA3 gene variant. Dig Liver Dis 2015;47:e231.
13. Chernyak V, Fowler KJ, Kamaya A, et al. Liver imaging reporting and data system (LI-RADS) version 2018: imaging of hepatocellular carcinoma in At-Risk patients. Radiology 2018;289:816–830.
14. Kirchmeyer M, Gaigneaux A, Servais FA, et al. Altered profiles of circulating cytokines in chronic liver diseases (NAFLD/HCC): impact of the PNPLA3I148M risk allele. Hepatol Commun 2023;7:e0306.
15. Margue C, Reinsbach S, Philippidou D, et al. Comparison of a healthy miRNome with melanoma patient miRNomes: are microRNAs suitable serum biomarkers for cancer? Oncotarget 2015;6:12110–12127.
16. Kassambara A. Rstatix: pipe-friendly framework for basic statistical tests, R Package Version 0.7.2. 2023. https://rpkgs.datanovia.com/rstatix/. Accessed March 9, 2025.
17. Benjamini Y, Hochberg Y. Controlling the false discovery rate: a practical and powerful approach to multiple testing. J R Stastistic Soc B 1995;57:289–300.

18. R Core Team. In: R: a language and environment for statistical computing. Vienna, Austria: R Foundation for Statistical Computing, 2022. https://www.R-project.org.
19. Wickham H. ggplot2: elegant graphics for data analysis. New York: Springer, 2016.
20. Wei T, Simko V. R package “corrplot”: visualization of a correlation matrix. vVersion 0.92. 2021. https://github.com/taiyun/corrplot. Accessed March 9, 2025.
21. Khan M, Brandenburger T. ROCit: performance assessment of binary classifier with visualization_. R Package. Version 2.1.1. 2020. https://CRAN.R-project.org/package=ROCit. Accessed March 9, 2025.
22. Kirchmeyer M, Servais FA, Hamdorf M, et al. Cytokine-mediated modulation of the hepatic miRNome: miR-146b-5p is an IL-6-inducible miRNA with multiple targets. J Leukoc Biol 2018;104:987–1002.
23. Fernandez-Tussy P, Fernandez-Ramos D, Lopitz-Otsoa F, et al. miR-873-5p targets mitochondrial GNMT-complex II interface contributing to non-alcoholic fatty liver disease. Mol Metab 2019;29:40–54.
24. Fernandez-Tussy P, Rodriguez-Agudo R, Fernandez-Ramos D, et al. Anti-miR-518d-5p overcomes liver tumor cell death resistance through mitochondrial activity. Cell Death Dis 2021;12:555.
25. Becker PP, Rau M, Schmitt J, et al. Performance of serum microRNAs -122, -192 and -21 as biomarkers in patients with non-alcoholic steatohepatitis. PLoS One 2015;10:e0142661.
26. Xu J, Wu C, Che X, et al. Circulating microRNAs, miR-21, miR-122, and miR-223, in patients with hepatocellular carcinoma or chronic hepatitis. Mol Carcinog 2011;50:136–142.
27. Pelizzaro F, Cardin R, Sartori A, et al. Circulating MicroRNA-21 and MicroRNA-122 as prognostic biomarkers in hepatocellular carcinoma patients treated with transarterial chemoembolization. Biomedicines 2021;9:890.
28. Sun C, Shi C, Duan X, et al. Exosomal microRNA-618 derived from mesenchymal stem cells attenuate the progression of hepatic fibrosis by targeting Smad4. Bioengineered 2022;13:5915–5927.
29. Zhang Q, Xu M, Qu Y, et al. Analysis of the differential expression of circulating microRNAs during the progression of hepatic fibrosis in patients with chronic hepatitis B virus infection. Mol Med Rep 2015;12:5647–5654.
30. Greco M, Mirabelli M, Salatino A, et al. From euglycemia to recent onset of type 2 diabetes mellitus: a proof-of-concept study on circulating microRNA profiling reveals distinct, and early microRNA signatures. Diagnostics (Basel) 2023;13:2443.
31. Li Y, Wang X, Li Z, et al. MicroRNA-4651 represses hepatocellular carcinoma cell growth and facilitates apoptosis via targeting FOXP4. Biosci Rep 2020;40:BSR20194011.
32. Zhang TQ, Su QQ, Huang XY, et al. Micro RNA-4651 serves as a potential biomarker for prognosis when selecting hepatocellular carcinoma patients for post-operative adjuvant transarterial chemoembolization therapy. Hepatol Commun 2018;2:1259–1273.
33. Elfert AY, Salem A, Abdelhamid AM, et al. Implication of miR-122, miR-483, and miR-335 expression levels as potential signatures in HCV-Related hepatocellular carcinoma (HCC) in Egyptian patients. Front Mol Biosci 2022;9:864839.
34. Cui L, Hu Y, Bai B, et al. Serum miR-335 level is associated with the treatment response to trans-arterial chemoembolization and prognosis in patients with hepatocellular carcinoma. Cell Physiol Biochem 2015;37:276–283.
35. Liu M, Liu X, Pan M, et al. Characterization and microRNA expression analysis of serum-derived extracellular vesicles in severe liver injury from chronic HBV infection. Life (Basel) 2023;13:347.
36. Thapa N, Chwae YJ, Yoo KH, et al. Exosomal delivery of TRAIL and miR-335 for the treatment of hepatocellular carcinoma (Review). Int J Mol Med 2023;51:3.
37. Yang X, Song H, Zi Z, et al. Circ_0005075 promotes hepatocellular carcinoma progression by suppression of microRNA-335. J Cell Physiol 2019;234:21937–21946.
38. Nie Y, Zhu X, Bu N, et al. Circ_0064288 acts as an oncogene of hepatocellular carcinoma cells by inhibiting miR-335-5p expression and promoting ROCK1 expression. BMC Cancer 2022;22:265.
39. Dohi O, Yasui K, Gen Y, et al. Epigenetic silencing of miR-335 and its host gene MEST in hepatocellular carcinoma. Int J Oncol 2013;42:411–418.
40. Wang F, Li L, Piontek K, et al. Exosome miR-335 as a novel therapeutic strategy in hepatocellular carcinoma. Hepatology 2018;67:940–954.
41. Zhang N, Hu Z, Qiang Y, et al. Circulating miR-130b- and miR-21-based diagnostic markers and therapeutic targets for hepatocellular carcinoma. Mol Genet Genomic Med 2019;7:e1012.
42. Wu J, Wu Y, Luo Y, et al. Circulating miRNA-199a and miRNA-122 levels as potential diagnostic and prognostic biomarkers for hepatocellular carcinoma. Ann Clin Lab Sci 2020;50:219–227.
43. Akuta N, Kawamura Y, Suzuki F, et al. Impact of circulating miR-122 for histological features and hepatocellular carcinoma of nonalcoholic fatty liver disease in Japan. Hepatol Int 2016;10:647–656.
44. Pirola CJ, Fernandez Gianotti T, Castano GO, et al. Circulating microRNA signature in non-alcoholic fatty liver disease: from serum non-coding RNAs to liver histology and disease pathogenesis. Gut 2015;64:800–812.
45. Hendy OM, Rabie H, El Fouly A, et al. The circulating Micro-RNAs (-122, -34a and -99a) as predictive biomarkers for non-alcoholic fatty liver diseases. Diabetes Metab Syndr Obes 2019;12:2715–2723.
46. Salvoza NC, Klinzing DC, Gopez-Cervantes J, et al. Association of circulating serum miR-34a and miR-122 with dyslipidemia among patients with non-alcoholic fatty liver disease. PLoS One 2016;11:e0153497.
47. Cermelli S, Ruggieri A, Marrero JA, et al. Circulating microRNAs in patients with chronic hepatitis C and non-alcoholic fatty liver disease. PLoS One 2011;6:e23937.
48. Zarrinpar A, Gupta S, Maurya MR, et al. Serum microRNAs explain discordance of non-alcoholic fatty liver disease in monozygotic and dizygotic twins: a prospective study. Gut 2016;65:1546–1554.

49. Celikbilek M, Baskol M, Taheri S, et al. Circulating microRNAs in patients with non-alcoholic fatty liver disease. World J Hepatol 2014;6:613–620.
50. Auguet T, Aragones G, Berlanga A, et al. miR33a/miR33b* and miR122 as possible contributors to hepatic lipid metabolism in Obese women with nonalcoholic fatty liver disease. Int J Mol Sci 2016;17:1620.
51. Kim TH, Lee Y, Lee YS, et al. Circulating miRNA is a useful diagnostic biomarker for nonalcoholic steatohepatitis in nonalcoholic fatty liver disease. Sci Rep 2021;11:14639.
52. Sun C, Huang F, Liu X, et al. miR-21 regulates triglyceride and cholesterol metabolism in non-alcoholic fatty liver disease by targeting HMGCR. Int J Mol Med 2015;35:847–853.
53. Pratama MY, Visintin A, Croce LS, et al. Circulatory miRNA as a biomarker for therapy response and disease-free survival in hepatocellular carcinoma. Cancers (Basel) 2020;12:2810.
54. Cai P, Mu Y, Olveda RM, et al. Circulating miRNAs as footprints for liver fibrosis grading in schistosomiasis. EBioMedicine 2018;37:334–343.
55. Blaya D, Pose E, Coll M, et al. Profiling circulating microRNAs in patients with cirrhosis and acute-on-chronic liver failure. JHEP Rep 2021;3:100233.
56. Soluyanova P, Quintas G, Perez-Rubio A, et al. The development of a non-invasive screening method based on serum microRNAs to quantify the percentage of liver steatosis. Biomolecules 2024;14:1423.
57. Kurdiova T, Balaz M, Kovanicova Z, et al. Serum afamin a novel marker of increased hepatic lipid content. Front Endocrinol (Lausanne) 2021;12:670425.
58. Ahn SB. Noninvasive serum biomarkers for liver steatosis in nonalcoholic fatty liver disease: current and future developments. Clin Mol Hepatol 2023;29:S150–S156.

Received June 27, 2025. Accepted October 24, 2025.

Correspondence:
Address correspondence to: Iris Behrmann, University of Luxembourg, 6, Avenue du Swing, 4367 Belvaux, Esch-sur-Alzette, Luxembourg. e-mail: iris.behrmann@uni.lu.

Acknowledgments:
The authors thank Prof. Stefan Rose-John (Christian-Albrechts-University, Kiel, Germany) for providing hyper-IL-6 and Dr Giulia Cesi for help with the graphical abstract.

Authors' Contributions:
Mélanie Kirchmeyer: Investigation, data analysis, writing (original draft). Anthoula Gaigneaux: Statistical data analysis, visualization, writing (original draft). Florence A. Servais: Investigation. Anita Arslanow: Resources, writing (review and editing). Claudia Rubie: Resources. Markus Casper: Resources, writing (review & editing). Matthias Glanemann: Resources. María L. Martínez-Chantar: Conceptualization, writing (review and editing). Marcin Krawczyk: Conceptualization, resources, writing (review & editing). Frank Lammert: Conceptualization, resources, writing (review and editing), funding acquisition. Iris Behrmann: Conceptualization, writing (original draft), supervision, funding acquisition.
All authors have approved the final version of the manuscript.

Conflict of Interest:
The authors disclose no conflicts.

Funding:
This work was funded by Fonds National de la Recherche Luxembourg, Deutsche Forschungsgemeinschaft (C12/BM/3975937, FL/997/7-1, Inter-project "HepmiRSTAT") and Bundesministerium für Forschung, Technologie und Raumfahrt (031L0051, 031L0256, 031L0257, 031L0313H LiSyM-Krebs).

Ethical Statement:
The sample collections were approved by the respective ethics committees in Germany (Ethik-Kommission der Ärztekammer des Saarlandes, 271/11–79/12) and Luxembourg (Comité National d'Éthique de Recherche, 201309/07). Written informed consent was obtained from all healthy controls and patients before inclusion in the study; the studies were performed in accordance with the Declaration of Helsinki and applicable national regulations.

Data Transparency Statement:
The data that support the findings of this study are available from the corresponding author upon reasonable request.

Reporting Guidelines:
Not applicable.

Gastro Hep Advances, Volume 5

Supplemental information

Identification of Circulating MiR-4651 as Novel Biomarker for Metabolic Dysfunction–Associated Steatotic Liver Disease

Mélanie Kirchmeyer, Anthoula Gaigneaux, Florence A. Servais, Anita Arslanow, Claudia Rubie, Markus Casper, Matthias Glanemann, María L. Martínez-Chantar, Marcin Krawczyk, Frank Lammert, and Iris Behrmann

Supplementary Information

Identification of Circulating MiR-4651 as Novel Biomarker for Metabolic Dysfunction-Associated Steatotic Liver Disease

Mélanie Kirchmeyer, Anthoula Gaigneaux, Florence A. Servais, Anita Arslanow, Claudia Rubie, Markus Casper, Matthias Glanemann, María L Martínez-Chantar, Marcin Krawczyk, Frank Lammert, Iris Behrmann

Table of content:

Supplementary Materials and Methods…………………………..…….……………..…….. p. 2
Supplementary References…..………………………………………….…………………… p. 2
Supplementary Figure A1……………..………………...……………………….……………… p. 3
Supplementary Figure A2………………………...…………….…………………………… p. 4
Supplementary Figure A3………………………………..…………………….…………….. p. 5
Supplementary Table A1…………………………………………………………….……….. p. 6

Supplementary Materials and Methods

qPCR Analysis of MiRNAs Isolated from Primary Human Liver Cells upon Stimulation with Hyper-IL-6

Primary healthy liver cells (named HepLux 5, 6, 7, and 8), derived from patients with colon cancer who underwent liver metastasis surgery, were received from the Department of Surgery of the Saarland University Hospital (Homburg, Germany). Human primary hepatocyte collection was approved by the respective ethics committees in Germany (Ethik-Kommission der Ärztekammer des Saarlands, 79/12) and Luxembourg (Comité National d'Éthique de Recherche, 201309/07), and signed statements of informed consent were obtained from all patients.

The handling and the culture of the cells have been described previously (1). Briefly, cells were maintained in HCM medium (HCM BulletKit Medium, Lonza, Basel, Switzerland) supplemented with 10% FBS and 100 µg/mL normocin. Six hours before the first stimulation, the HCM medium was replaced by serum-free hepatocyte high performance medium (Upcyte technologies, Hamburg, Germany), then kept untreated (Ct) or stimulated with 20 ng/mL hyper-IL-6 (a "designer cytokine" comprising IL-6 linked to the extracellular domain of IL-6Rα, (2)) for 6, 24, 48, and 72 h. Total RNA was extracted using the Column-zol RNA isolation kit (Enzymax LLC, Lexington, Kentucky, USA) according to the manufacturer's instructions. 500 ng of total RNA was reverse-transcribed and qPCR for miR-4651 and miR-4530 was run with QuantiTech primers (Qiagen, Venlo, The Netherlands), as well as for small RNAs used as miRNA normalizers (3 out of RNU1A, RNU5A, SCARNA17, and SNORD95).

Supplementary References

1. Kirchmeyer M, Servais FA, Hamdorf M, et al. Cytokine-mediated modulation of the hepatic miRNome: miR-146b-5p is an IL-6-inducible miRNA with multiple targets. J Leukoc Biol 2018;104:987-1002.
2. Fischer M, Goldschmitt J, Peschel C, et al. I. A bioactive designer cytokine for human hematopoietic progenitor cell expansion. Nat Biotechnol 1997;15:142-145.
3. Kirchmeyer M, Gaigneaux A, Servais FA, et al. Altered profiles of circulating cytokines in chronic liver diseases (NAFLD/HCC): Impact of the PNPLA3I148M risk allele. Hepatol Commun 2023;7.

Supplementary Figure A1

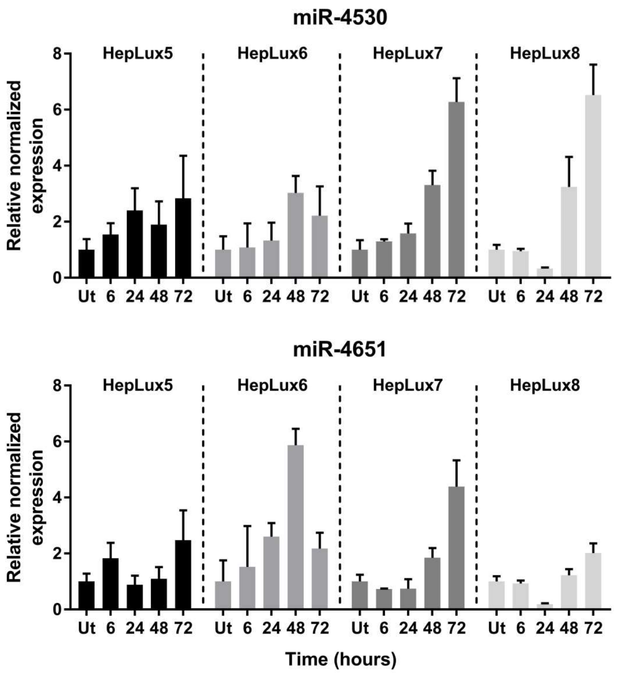

Supplementary Figure A1: Inducible expression of miR-4530 and miR-4651 in primary human hepatocytes treated with hyper-IL-6. Primary hepatocytes isolated from four patients (HepLux5-8) were stimulated with 20 ng/mL hyper-IL-6 for 6 - 72 h or left untreated (Ut). The levels of miR-4530 and miR-4651 in RNA extracts were determined by qPCR; results were normalized to the control. Error bars indicate the SD based on two technical replicates for the 6, 24, and 48 h time points, and three replicates for the Ut and 72 h conditions, all performed in parallel. (Please note: for HepLux8, one of the two 24 h-samples showed aberrant profiles and was therefore removed).

Supplementary Figure A2

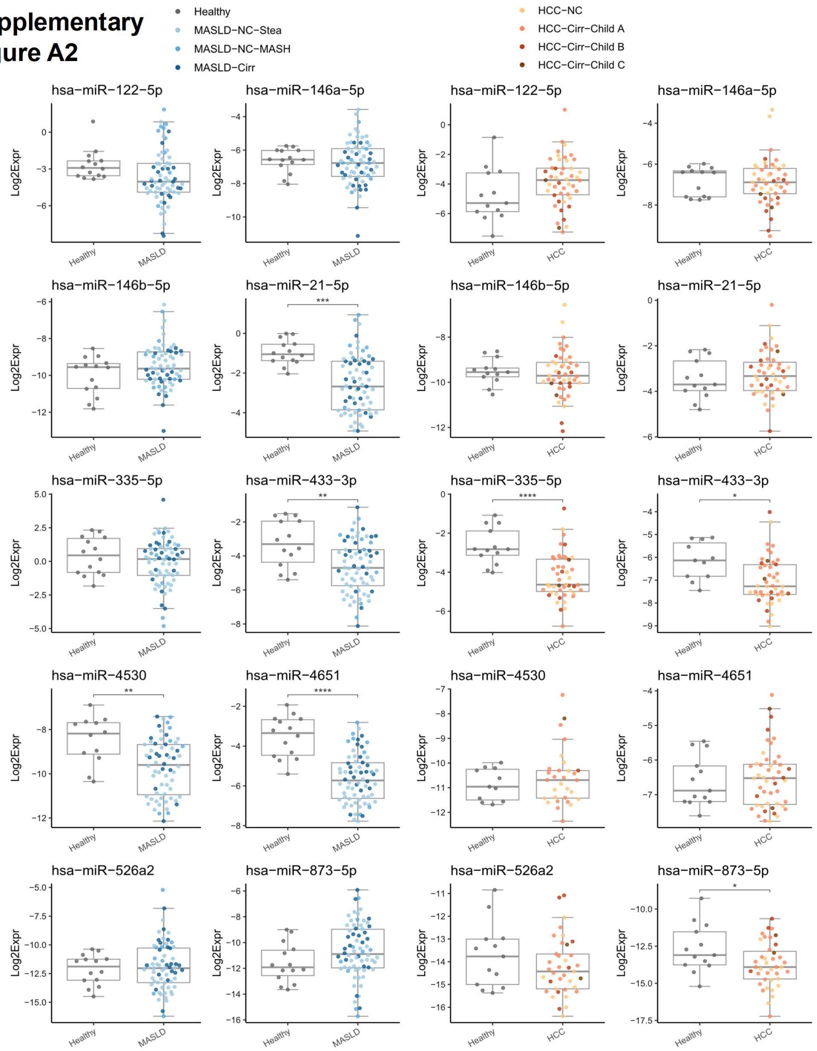

Supplementary Figure A2: Profiles of the 10 miRNAs in patients with MASLD and HCC *vs*. healthy controls. NAFLD and HCC samples were analyzed without further subdivision in disease-subtypes (in contrast to Figure 1). The horizontal line within the box plots represents the median, vertical lines from the boxes (whiskers) indicate the variability outside the upper and lower quartiles. Pairwise t-tests were performed, and p-values were adjusted for each miRNA using the Holm method. ****: $p < 0.0001$, ***: $p < 0.001$, **: $p < 0.01$, *: $p < 0.05$.

Supplementary Figure A3

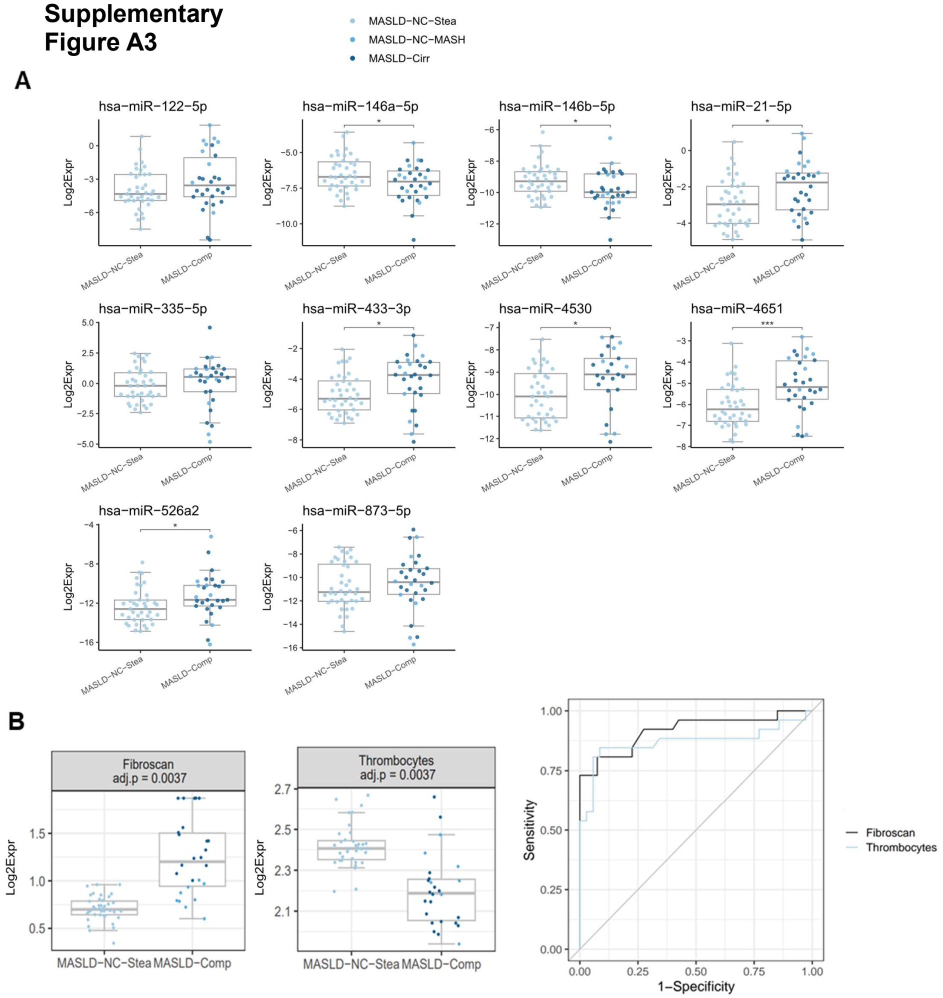

Supplementary Figure A3: Comparison of patients with uncomplicated MASLD (MASLD-NC-Stea, i.e., simple steatosis) *vs*. complicated MASLD (MASLD-Comp, i.e., MASH and cirrhosis). A) Profiles of the 10 miRNAs. B) Single-parameter ROC curves for “Fibroscan” and “Thrombocytes” (see also Figure 5 and Table S1). The BH-adjusted p-value of the GLM model is indicated.

Supplementary Table A1: Information about clinical parameters and data availability.

Parameters	Healthy	MASLD-NC-Stea	MASLD-NC-MASH	MASLD-Cirr	HCC-NC	HCC-Cirr	Overall
	(N=14)	(N=40)	(N=9)	(N=21)	(N=15)	(N=32)	(N=131*)
**Thrombocytes (10^9/L)**							
Mean (SD)	225 (58.4)	271 (71.6)	191 (67.3)	166 (92.2)	/	/	228 (85.8)
Median	216	255	177	141	/	/	223
[Min, Max]	[157, 338]	[157, 467]	[87.0, 298]	[97.0, 458]			[87.0, 467]
Missing	0 (0%)	5 (12.5%)	2 (22.2%)	2 (9.5%)			9 (10.7%*)
**GGT (U/L)**							
Mean (SD)	27.1 (22.8)	72.3 (73.9)	327 (440)	134 (105)	169 (144)	301 (423)	194 (304)
Median	19.5	39.0	154	125	121	200	109
[Min, Max]	[7.00, 97.0]	[21.0, 157]	[58.0, 1200]	[26.0, 438]	[24.0, 530]	[25.0, 2260]	[7.00, 2260]
Missing	0 (0%)	37 (92.5%)	3 (33.3%)	5 (23.8%)	1 (6.7%)	2 (6.3%)	48 (36.6%)
**AST (U/L)**							
Mean (SD)	20.1 (5.53)	32.3 (12.5)	92.3 (58.5)	46.0 (39.6)	39.2 (13.6)	63.7 (43.9)	49.2 (40.2)
Median	19.0	38.0	91.0	33.0	36.0	49.0	36.0
[Min, Max]	[12.0, 32.0]	[18.0, 41.0]	[32.0, 183]	[21.0, 173]	[20.0, 61.0]	[26.0, 205]	[12.0, 205]
Missing	1 (7.1%)	37 (92.5%)	3 (33.3%)	5 (23.8%)	4 (26.7%)	9 (28.1%)	59 (45.0%)
**Cytokine data**							
Available	11 (78.6%)	40 (100%)	8 (88.9%)	21 (100%)	6 (40.0%)	15 (46.9%)	101 (77.1%)
Missing	3 (21.4%)	0 (0%)	1 (11.1%)	0 (0%)	9 (60.0%)	17 (53.1%)	30 (22.9%)

For the six groups (Healthy, MASLD-NC-Stea, MASLD-NC-MASH, MASLD-Cirr, HCC-NC, HCC-Cirr), information about the clinical parameters “thrombocytes”, GGT, and AST are indicated as mean ± SD and median. Moreover, the numbers of samples analyzed previously regarding concentrations of 22 cytokines (3) are indicated as well as the numbers of missing values.

*For “thrombocytes”, no data were collected for HCC patients. Thus, the percentage was calculated only for the healthy and MASLD samples (N=84).
